# Multiple biochemical indices and metabolomics of *Clonorchis sinensis* provide a novel interpretation of biomarkers

**DOI:** 10.1186/s13071-022-05290-y

**Published:** 2022-05-19

**Authors:** Yang-Yuan Qiu, Qiao-Cheng Chang, Jun-Feng Gao, Ming-Jia Bao, Hai-Tao Luo, Jin-Ho Song, Sung-Jong Hong, Rui-Feng Mao, Yun-Yi Sun, Ying-Yu Chen, Ming-Yuan Liu, Chun-Ren Wang, Xiao-Lei Liu

**Affiliations:** 1 College of Animal Science and Veterinary Medicine, Heilongjiang Bayi Agricultural University, Daqing, 163319 Heilongjiang Province China; 2 Key Laboratory of Zoonosis Research, Ministry of Education, Institute of Zoonosis, College of Veterinary Medicine, Jilin University, Changchun, 130012 Jilin Province China; 3 School of Public Health, Shantou University, Shantou, 515063 Guangdong Province China; 4 Jiamusi Center for Disease Control and Prevention, Jiamusi, 154000 Heilongjiang Province China; 5 Department of Clinical Laboratory, Qiqihar Traditional Chinese Medicine Hospital, Qiqihar, 161000 Heilongjiang Province China; 6 Department of Pharmacology, Chung-Ang University College of Medicine, Seoul, 06974 Republic of Korea; 7 Convergence Research Center for Insect Vectors, Incheon National University, Incheon, 22012 Republic of Korea

**Keywords:** *Clonorchis sinensis*, Plasma metabolomics, Biochemical indices, Biomarkers, Amino acid metabolism

## Abstract

**Background:**

Clonorchiasis, an infectious disease caused by the liver fluke *Clonorchis sinensis*, may lead to the development of liver and gallbladder diseases, and even cholangiocarcinoma (CCA). However, the pathogenesis, host-pathogen interaction, and diagnostic markers for clonorchiasis remain unclear.

**Methods:**

Eighteen rabbits were randomly divided into control group (*n* = 9) and *C. sinensis*-infected group (*n* = 9), and their plasma samples were collected at 7, 14, 28, and 63 days post-infection (dpi). Biochemical indices and metabolites in different infection periods were detected. A non-targeted ultra-performance liquid chromatography-tandem mass spectrometry (UPLC-MS/MS) approach was employed to investigate the metabolic profiles of plasma in rabbits, and related metabolic pathways of differential metabolites and correlation between candidate biochemical indices and differential metabolites were analyzed. Finally, the candidate biomarkers were verified with human samples using a targeted metabolomics method.

**Results:**

The result of biochemical indices indicated *C. sinensis* infection would affect the liver function biochemical indices, especially alanine aminotransferase, aspartate transaminase (AST), glutamyl transpeptidase (GGT), total bile acid, high-density lipoprotein, and cholinesterase. The metabonomic results showed that 58, 212, 23, and 21 differential metabolites were identified in different phases of the infection. Multivariate statistical analysis of differential metabolites revealed distinct metabolic signatures during different phases of infection, with most of these signatures being observed at 14 dpi, which mainly influences the amino acid metabolisms. For metabolites and biochemical indices, AST, GGT, hypoxanthine, l-pipecolic acid, and d-glucuronate represented potential noninvasive biomarkers for the diagnosis of *C. sinensis* (*P* < 0.05 and AUC > 0.8). Furthermore, GGT and d-glucuronate levels were positively correlated with the infection (*r*(28) = 0.98, *P* < 0.0001) and showed excellent diagnostic performance (AUC = 0.972; 95% confidence interval, 0.921 to 1.000).

**Conclusions:**

The present results provide new insights into plasma metabolic changes in rabbits during *C. sinensis* infection, and the potential biomarker may be used for developing an effective method to diagnose clonorchiasis in the future.

**Graphical Abstract:**

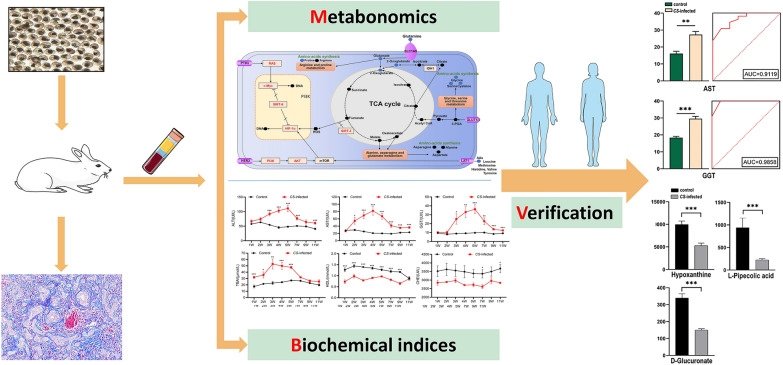

**Supplementary Information:**

The online version contains supplementary material available at 10.1186/s13071-022-05290-y.

## Background

Fish-borne zoonotic trematodes are the causative agents of fluke diseases in animals (such as dogs and cats) and humans. Clonorchiasis, caused by *Clonorchis sinensis*, is regarded as a major parasitic zoonosis in China [1]. The average prevalence of *C. sinensis* infection was estimated to be 20.5% and 41.8% in dogs and cats, respectively, in subtropical southern China between October 2006 and March 2008 [2]. In addition, it is estimated that approximately 200 million people are at the risk of *C. sinensis* infection, and it is estimated that 15 million people have been infected with *C. sinensis* worldwide, including China, South Korea, northern Vietnam, and far-eastern Russia [3, 4]. Approximately 5000 cholangiocarcinoma (CCA) cases attributed to *C. sinensis* infection are predicted to occur annually in the world in the coming decades [5]. *Clonorchis sinensis* was classified as a Group I biological carcinogen by the International Agency for Research on Cancer (IARC) of the World Health Organization in 2009 [6]. Thus, *C. sinensis* is a significant biological risk factor for human health in epidemic areas.

Clonorchiasis can be diagnosed using various methods including microscopic detection of eggs in fecal samples, polymerase chain reaction techniques, immunological techniques, and ultrasound imaging [4]. Egg detection in fecal samples is the most commonly used diagnostic approach [7]. However, eggs of certain trematode species, such as *Metorchis orientalis*, are similar to those of *C. sinensis* [8]. In addition, the analysis of certain biochemical indices has revealed that alanine aminotransferase (ALT), aspartate transaminase (AST), alkaline phosphatase (ALP), and glutamyl transpeptidase (GGT) may be used as early diagnostic markers [9]. Although studies have focused on the diagnosis, epidemiology, pathological mechanism, treatment, and vaccination for the disease [4], the diagnostic markers and molecular mechanisms remain poorly understood. Omics play an important role in screening the diagnostic markers of differential genes, proteins, and metabolites; the development of drug and vaccine targets; and elucidating the pathogenesis [10–12]. Metabolomics is a post-genomic discipline for studying the metabolome, and it can specifically reflect the real-time status of an organism, which makes it an optimal “readout” for external stimuli [13]. Ghosh et al. demonstrated the possibility of differentiating and identifying animals with cerebral malaria at an early pre-clinical stage based on serum metabolite profiles [14]. A non-targeted metabolomics approach has been shown to reveal the perturbations of spleen metabolism in clonorchiasis cases [12]. However, an effective and noninvasive biomarker has not been reported for the diagnosis of *C. sinensis* infections yet.

In clinical practice, the earlier a disease is diagnosed, the sooner it can be treated. Metabolites and biochemical indices are the most intuitive components of response to diseases, and a combination of the two may provide more information. Thus, we report the plasma metabolomics of *C. sinensis* infection and the role of differential molecules in the crosstalk between metabolites and biochemical indices in disease progression of clonorchiasis.

## Methods

### Rabbit experiments

Metacercariae of *C. sinensis* were collected from muscular tissues of naturally infected *Pseudorasbora parva* in the endemic area of Qiqihar, Heilongjiang Province, China. The muscular tissue was digested with artificial digestive juice (1% pepsin-hydrochloric acid; Aladdin, China), and viable metacercariae were identified and collected via microscopy (CX23, Olympus, Japan). Japanese White rabbits (*n* = 18; weighing 2000–2250 g) were purchased from Changchun Yisi Experimental Animal Biotech Co. (SCXK-2016–0004) and randomly allocated into two groups: *C. sinensis*-infected group (500 metacercariae/rabbit; *n* = 9) and control group [treated with 500 μl phosphate-buffered saline (PBS); *n* = 9]. The rabbits were housed in separate cages at a controlled temperature (22 ± 2 °C; 12-h light/dark cycle) and were provided with water and pellet feed ad libitum. Plasma samples were collected from the ear veins of the rabbits after weeks 1, 2, 3, 4, 5, 6, 7, 9, and 11. The samples were stored at –80 °C after inactivation with liquid nitrogen. All animal studies were performed in accordance with the Guide for the Care and Use of Laboratory Animals (1996).

Fecal samples of the rabbits in the treated group were observed under the microscope to confirm *C. sinensis* infection. Subsequently, the rabbits were anesthetized using isoflurane and killed by injecting air through ear vein at 77 days post-infection. Following the rapid isolation of liver in an aseptic environment, adult parasites were collected from the liver and bile duct and identified through internal transcribed spacer (ITS) sequencing (forward primer: 5ʹ- GTA GGT GAA CCT GCG GAA GGA TCA TT -3ʹ; reverse primer: 5ʹ- TTA GTT TCT TTT CCT CCG CT -3ʹ). The liver tissue was then cut into small pieces, rinsed with saline solution (0.9% NaCl w/v), stored in 10% neutral buffered formalin for 1 week, dehydrated in ethanol, and embedded in paraffin wax. Five-micron paraffin sections were stained with hematoxylin and eosin (H&E) and Masson’s trichrome staining and examined under a light microscope (CX23, Olympus, Japan).

### Detection of biochemical indices

An automatic biochemical analyzer (AU5800, Beckman Coulter, USA) was used to detect total protein (TP), albumin (ALB), globulin (GLB), total bile acid (TBA), ALT, AST, GGT, lactate dehydrogenase (LDH), cholesterol (CHOL), triglyceride (TG), high density lipoprotein (HDL), low density lipoprotein (LDL), blood urea nitrogen (BUN), creatinine (CREA), uric acid (UA), glucose (GLU), prealbumin (PA), and cholinesterase (CHE) content in the samples.

### Metabolite extraction

Plasma samples were thawed at 4 ℃ on ice, and 100 μl of sample was taken in an EP tube and extracted with 400 μl of extraction solvent (V_methanol_:V_acetonitrile_ = 1:1) containing internal standard (2-chloro-l-phenylalanine, 2 μg/ml). This was vortex-mixed for 30 s and sonicated for 10 min (incubated in ice water), and the proteins were precipitated at − 20 ℃ for 1 h; 500 μl liquid was centrifuged at 12,000 rpm for 15 min at 4 ℃, and 425 μl supernatant was transferred into EP tubes. The supernatant was dried in a vacuum concentrator without heating, reconstituted with 100 μl extraction solvent (V_acetonitrile_:V_water_ = 1:1), vortex mixed for 30 s, sonicated for 10 min (4 ℃ water bath), and centrifuged at 12,000 rpm for 15 min at 4 ℃. Finally, 60 μl of the supernatant was used for ultra-high performance liquid chromatography-quadrupole time-of-flight mass spectrometry analysis (UHPLC-QTOF-MS). For monitoring the performance of data acquisition, 11 quality control (QC) samples were prepared using 10 μl from each sample.

### Liquid chromatography-tandem mass spectrometry (LC-MS/MS) analysis

LC-MS/MS assay was performed using a UHPLC system (1290, Agilent Technologies) with an UPLC BEH Amide column (1.7 μm, 2.1 × 100 mm, Waters) connected to a Triple TOF 6600® system (Q-TOF, AB SCIEX). The mobile phase consisting of 25 mM NH_4_CH_3_CO_2_ and 25 mM NH_4_OH in water (pH = 9.75) (A) and acetonitrile (B) was carried out with elution gradient as follows: 0 min, 95% B; 0.5 min, 95% B; 7 min, 65% B; 8 min, 40% B; 9 min, 40% B; 9.1 min, 95% B; 12 min, 95% B, delivered at 0.5 ml/min. The injection volume was 1.5 μl. The Triple TOF mass spectrometer was used to acquire MS/MS spectra on an information-dependent acquisition basis during an the LC/MS experiment. In this mode, the acquisition software (Analyst TF 1.7, AB SCIEX) continuously evaluates the full scan survey MS data as it collects and triggers the acquisition of MS/MS spectra depending on preselected criteria. In each cycle, 12 precursor ions having intensity > 100 were chosen for fragmentation at a collision energy of 30 V (15 MS/MS events with product ion accumulation time of 50 ms each). Electrospray ionization (ESI) source conditions were set as follows: ion source gas 1 at 60 Psi, ion source gas 2 at 60 Psi, curtain gas at 35 Psi, source temperature at 650 ℃, and ion spray voltage floating at 5000 V in positive and − 4000 V in negative modes, respectively.

### Identification of the metabolites and data analysis

MS raw data (.wiff) files were converted to the mzXML format using ProteoWizard and processed by the R package XCMS (version 3.2). The preprocessing results generated a data matrix that consisted of the retention time, mass-to-charge ratio (m/z) values, and peak intensity. The R package CAMERA was used for peak annotation after XCMS data processing. In-house MS2 database was developed to identify metabolites assisted by Biotree Biotech Co., Ltd. (Shanghai, China).

The resultant data set was uploaded to the SIMCA (version 14.1, Umetrics, Umea, Sweden) for principal component analysis (PCA) and orthogonal partial least squares discriminant analysis (OPLS-DA). The data for both negative and positive ion modes were mean-centered and scaled using Unit-Variance (for PCA) or Pareto (for OPLS-DA) before multivariate statistical analysis. Differential metabolites were identified by variable importance in projection (VIP) values > 1 in OPLS-DA model and Student’s *t*-test on the normalized peak areas (*P* < 0.05). To check the distinctions in metabolic state of the *C. sinensis*-infected group in different infection periods, log_2_ transformation was performed for cluster analysis, and heat maps were generated using Multiple Experiment Viewer 4.9.0 (http://mev.tm4.org/). Metabolite pathways were analyzed using Kyoto Encyclopedia of Genes and Genomes (KEGG, https://www.kegg.jp/kegg/) database and MetaboAnalyst (http://www.metaboanalyst.ca/).

### Detection of candidate biomarkers

To identify the potential biochemical indices and metabolites for clinical diagnosis, serum samples of patients were collected from *C. sinensis*-endemic areas in Jilin Province, China. Patients having chronic diseases (such as diabetes and high blood pressure) and other hepatic diseases (not caused by *C. sinensis*) were excluded. Samples from 8 healthy people and 22 with *C. sinensis* infection were collected. Biochemical indices were detected using an automatic biochemical analyzer (AU5800 Beckman Coulter, USA), and the targeted metabolites were detected by internal standard method. Metabolite extraction and detection were based on the previous literature [15]. Six standards included glycodeoxycholic acid (CAS: 360–65-6, Macklin, China), xanthine (CAS: 69-89-6, Macklin, China), hypoxanthine (CAS: 68-94-0, Acmec, China), d-glucuronate (CAS: 12-3-6556, Sigma-Aldrich, St Louis, MO), l-pipecolic acid (CAS: 3105–95-1, Macklin, China), and 3-methylglutaric acid (CAS: 626-51-7, Macklin, China). Additionally, two internal standards were used: naptalam (CAS: 132-66-1, Sigma-Aldrich, St Louis, MO) and tinidazole (CAS: 19387-91-8, Macklin, China). The primary and secondary MS data were collected under MRM mode of Analyst 1.7.0 software of the AB SCIEX 4500 mass spectrometer (AB SCIEX).

### Correlation between candidate biochemical indices and metabolites

The correlation between candidate biochemical indices and metabolites was analyzed using Spearman’s correlation coefficient (https://hiplot.com.cn/) analysis. Binary logistic regression and receiver-operating characteristic (ROC) curve analyses were performed to identify biomarkers for clonorchiasis. The area under the curve (AUC) was used to assess the diagnostic accuracy: 0.8 < AUC < 0.9 as good and 0.9 < AUC ≤ 1.0 as excellent.

### Statistical analysis

SPSS Statistics software version 22 (IBM, Armonk, NY, USA) was used for statistical analysis of the data, and Prism version 8.0.1 (GraphPad Software, San Diego, CA) was used for generating plots. Statistical analysis of every biochemical index and metabolite analyzed during each infection period was performed using unpaired two-tailed Student’s *t*-test (*P < 0.05, **P < 0.01, ***P < 0.001) between *C. sinensis*-infected and control groups. Results are expressed as mean ± SEM.

## Results

### Animal infection and histopathological analysis

*Clonorchis sinensis* eggs were first identified during a fecal examination at 19 dpi and confirmed in all infected rabbits at 25 dpi (Additional file 1: Fig. S1a). The egg ITS sequences were amplified (Additional file 1: Fig. S1b), and they showed 99.8% similarity with the published sequence (KF740423.1, China strains). H&E and Masson’s trichrome staining of rabbit liver tissues in the *C. sinensis*-infected group showed a disordered structure of hepatic lobules, severe hyperplasia of the bile duct, a large area of collagen fiber hyperplasia, and an increase in lymphocyte and neutrophil infiltration (Additional file 1: Fig. S1c, d).

### Biochemical indices detection

**Fig. 1:**
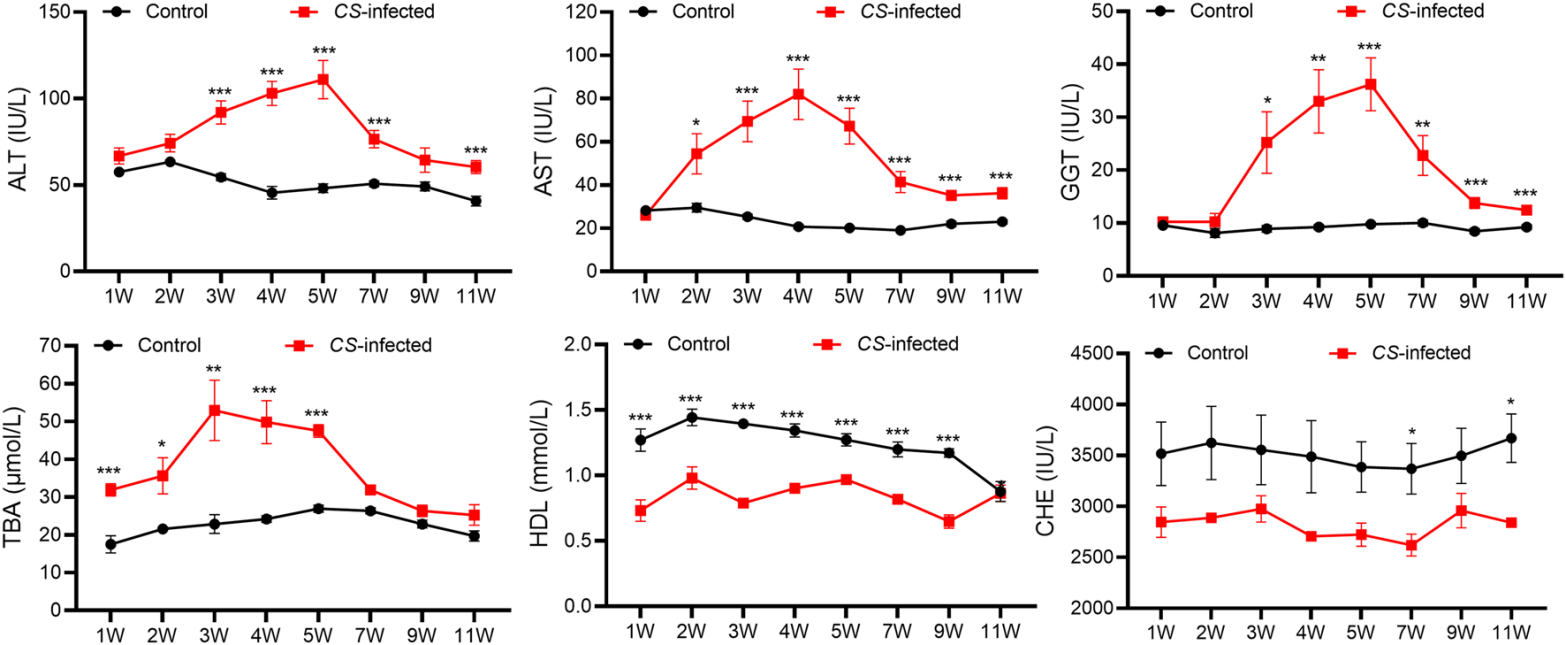
Changes in biochemical indices at different periods of *Clonorchis sinensis* infection. ALT, alanine aminotransferase; AST, aspartate transaminase; GGT, glutamyl transpeptidase; TBA, total bile acid; HDL, high density lipoprotein; CHE, cholinesterase; dpi, days post-infection. *P < 0.05, **P < 0.01, ***P < 0.001, *C. sinensis*-infected group (*n* = 9) compared to the control group (*n* = 9)

The serum samples collected at eight different periods of infection were used to analyze 19 different biochemical indices. The data of specific biochemical indicators for different groups at different periods are shown in Table S1 (Additional file 2). Several biochemical indices showed significant differences between the two groups (Additional file 1: Fig. S2). Specific biochemical indices showing apparent changes are presented in Fig. 1. TBA, ALT, AST, and GGT increased, whereas HDL and CHE decreased in the *C. sinensis*-infected group. Notably, changes in TBA, ALT, AST, and GGT during the experimental period of 11 weeks showed a bell-shaped pattern with peaks at 3–5 weeks (Fig. 1).

### Metabolite profiles

The data obtained from negative and positive ESI modes (ESI− and ESI+) were analyzed using PCA. The PCA of the samples including QC samples demonstrated that the data of all samples were within a 95% confidence interval (CI) based on Hotelling's T-squared distribution, and all QC samples were distributed within a twofold standard error, indicating that the quality of the data was very competent (Additional file 1: Figure S3).

**Fig. 2:**
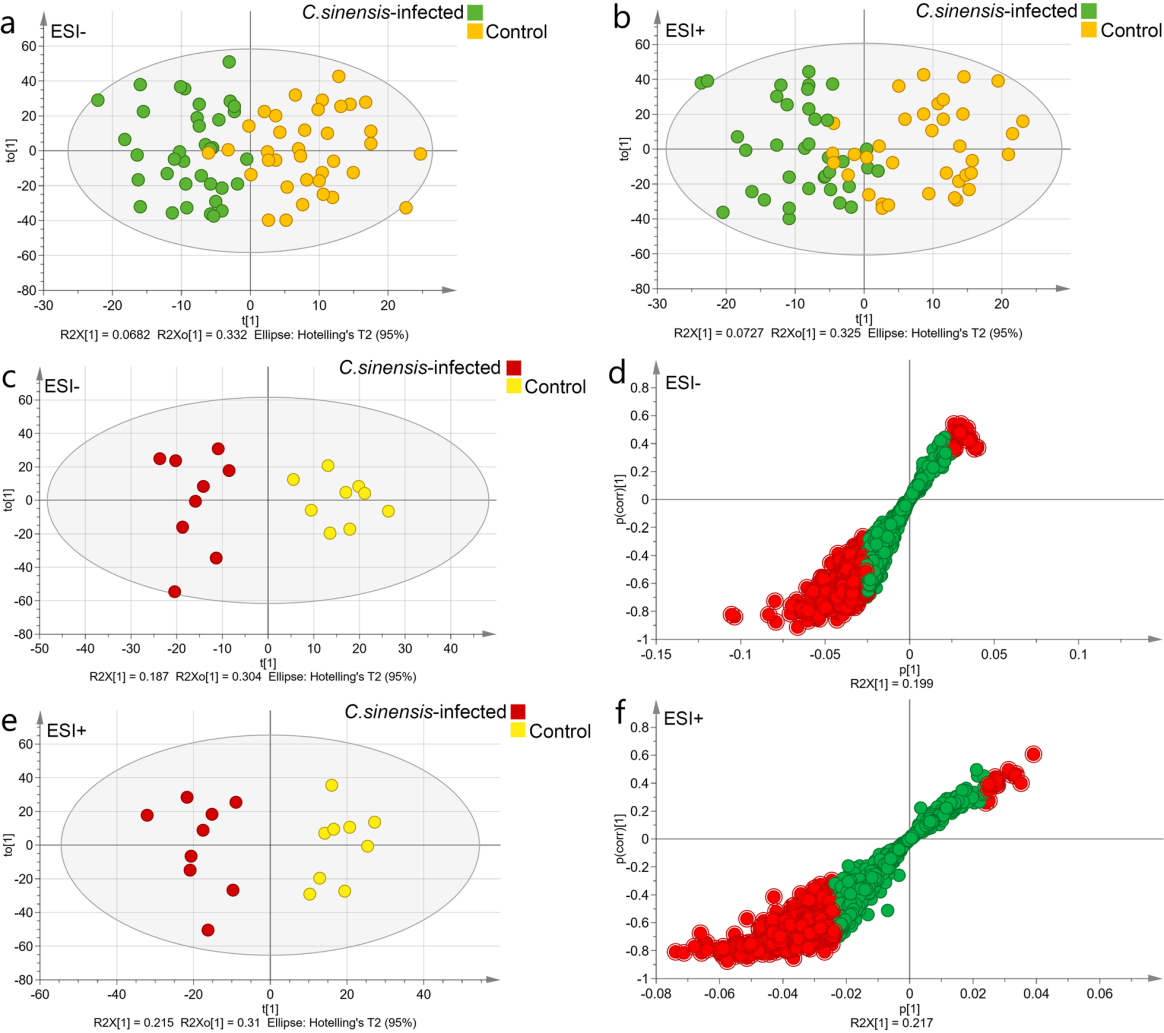
Scatter plots and loading plots for the predicted scores of the two components retained in the orthogonal partial least squares discriminant analysis (OPLS-DA) models for both negative electrospray ionization (ESI−) and positive (ESI+) electrospray ionization. **a** OPLS-DA model of metabolites in the different infection phases under ESI− mode. **b** OPLS-DA model of metabolites in the different infection phases under ESI+ mode. **c** OPLS-DA model of metabolites at 14 days post-infection (dpi) under ESI− mode. **d** Loading plot of OPLS-DA model of metabolites at 14 dpi under ESI− mode; red circle represents variable importance in projection (VIP) value > 1. **e** OPLS-DA model of metabolites at 14 dpi under ESI+ mode. **f** Loading plot of OPLS-DA model of metabolites at 14 dpi under ESI+ mode. Red circle represents differential metabolites and VIP value > 1

The OPLS-DA showed a clear distinction between metabolite data sets of all *C. sinensis*-infected and control group samples (Fig. 2a, b). Differential metabolites were identified at 7, 14, 28, and 63 dpi under different modes, and 14 dpi had the maximum differential metabolites (Additional file 2: Tables S2–S5). As shown in Fig. 2c–f, the OPLS-DA model made an efficient interpretation and identified different metabolites at 14 dpi.

### Relationship of the metabolic pathways during different infection phases

**Fig. 3:**
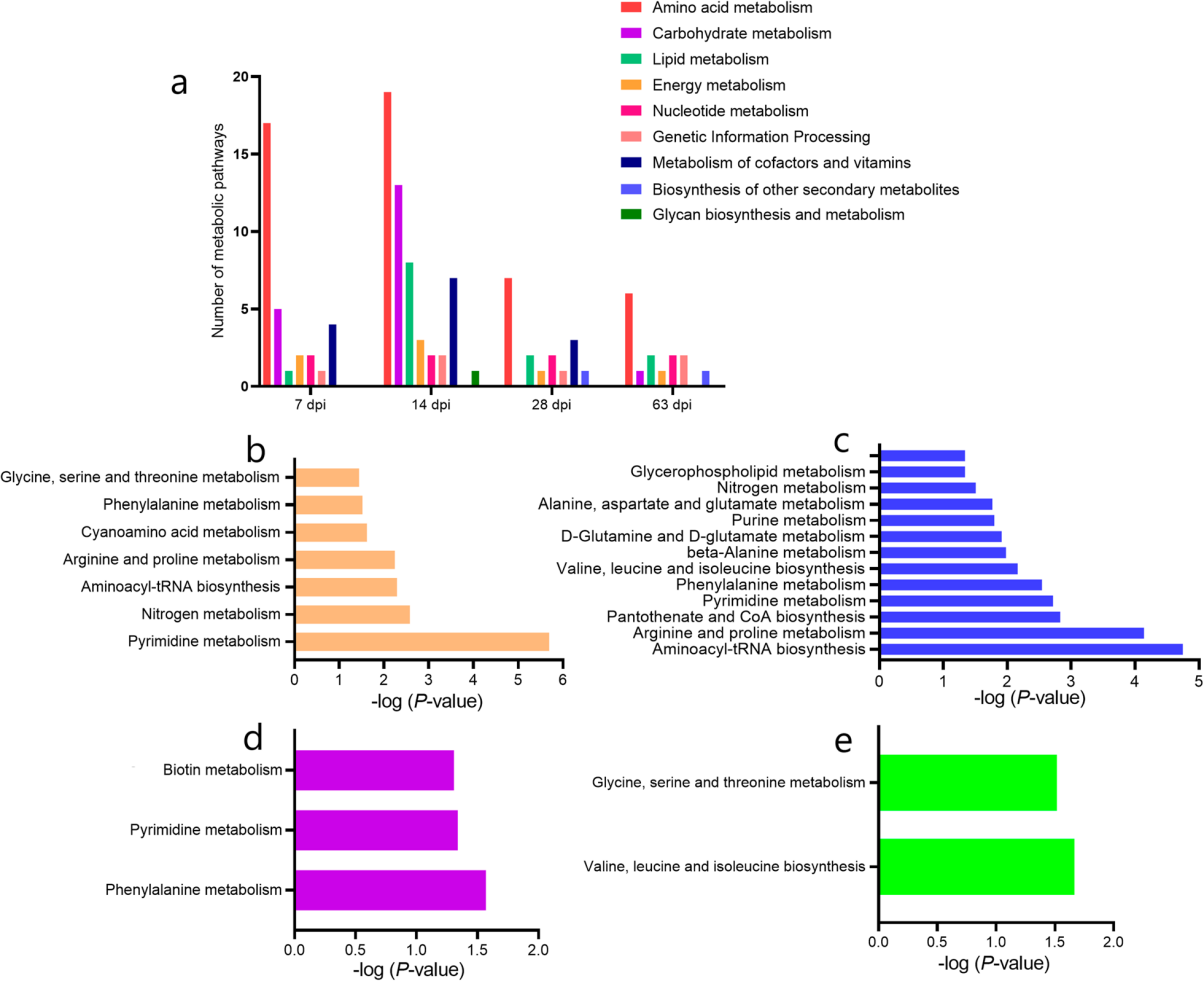
Metabolic pathways of differential metabolite enrichment in the rabbits infected with *Clonorchis sinensis* at different periods. **a** Classification of metabolic pathways in four periods [7 days post-infection (dpi), 14 dpi, 28 dpi and 63 dpi]. Specific pathways at **b** 7 dpi. **c** 14 dpi. **d** 28 dpi. **e** 63 dpi

**Fig. 4:**
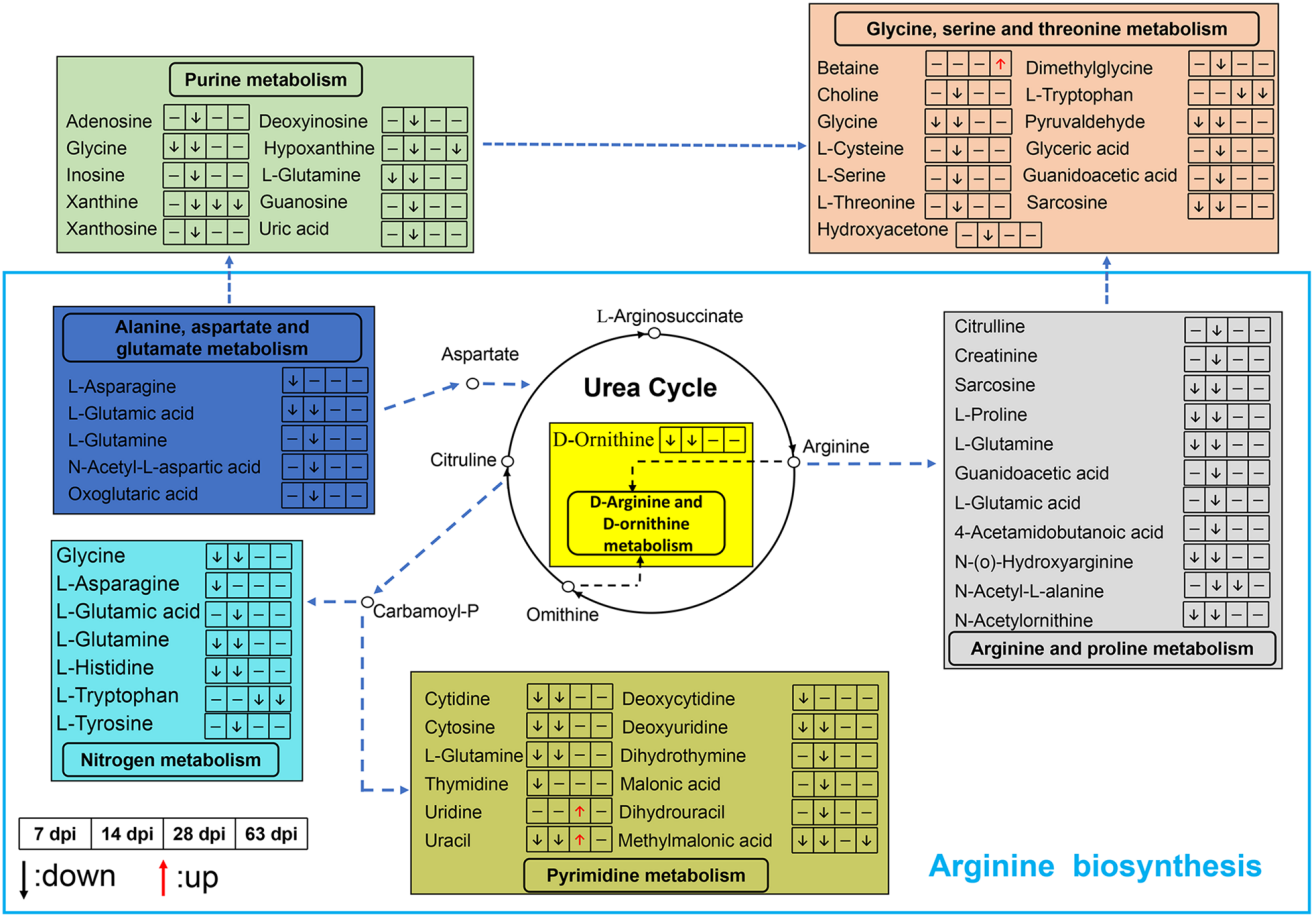
Correlation analysis of differential metabolites therein under four different periods. Different background colors represent different metabolic pathways; ↓, downregulated differential metabolite; ↑, upregulated differential metabolite

The most significantly perturbed metabolic pathways and differential metabolites in each perturbed metabolic pathway are shown in Table 1. Further analysis showed the most common metabolic pathway was related to amino acid metabolism (Fig. 3a). Among the pathways (Additional file 2: Table S6), 15 metabolic pathways were significant (*P* < 0.05) in four periods (Fig. 3b–e). Furthermore, metabolic pathways in different stages were found to be connected (Fig. 4). Different metabolic pathways were activated at various stages, and the metabolic pathways at 7 and 14 dpi were substantially activated compared with those at other infection stages. In addition, at 14 dpi, the pathway of central carbon metabolism in cancer was found to be activated according to the KEGG database (Additional file 1: Figure S4).

**Table 1:** Analysis of top five metabolic pathways via MetaboAnalyst based on all identified differential metabolites in four phases

Pathway name	Compound hits	*P*-value	Pathway impact	Metabolites
7 dpi				
Pyrimidine metabolism	8/60	2.03E−06	0.199	l-glutamine; cytidine; cytosine; uracil; deoxycytidine; deoxyuridine; thymidine; methylmalonic acid
Nitrogen metabolism	4/39	2.65E−03	0.008	l-asparagine; l-glutamine; l-histidine; glycine
Aminoacyl-tRNA biosynthesis	5/75	5.13E−03	0	l-asparagine; l-histidine; l-glutamine; glycine; l-proline
Arginine and proline metabolism	5/77	5.75E−03	0.120	l-glutamine; *N*-acetylornithine; l-proline; *N*-(o)-hydroxyarginine; sarcosine
Cyanoamino acid metabolism	2/16	2.41E−02	0	l-asparagine; glycine
14 dpi				
Glycine, serine, and threonine metabolism	11/48	1.79E−05	0.511	l-serine; choline; glyceric acid; guanidoacetic acid; dimethylglycine; glycine; sarcosine; l-threonine; pyruvaldehyde; l-cysteine; hydroxyacetone
Aminoacyl-tRNA biosynthesis	13/75	7.28E−05	0.169	l-histidine; l-glutamine; l-cysteine; glycine; l-serine; l-methionine; l-valine; l-lysine; l-leucine; l-threonine; l-tyrosine; l-proline; l-glutamic acid
Arginine and proline metabolism	11/77	1.48E−03	0.218	l-glutamine; citrulline; l-glutamic acid; *N*-acetyl-l-alanine; *N*-acetylornithine; l-proline; *N*-(o)-hydroxyarginine; guanidoacetic acid; creatinine; 4-acetamidobutanoic acid; sarcosine
Pantothenate and CoA biosynthesis	6/27	1.92E−03	0.274	l-cysteine; pantothenic acid; dihydrouracil; alpha-ketoisovaleric acid; l-valine; uracil
Pyrimidine metabolism	9/60	2.86E−03	0.174	l-glutamine; dihydrouracil; cytidine; cytosine; uracil; deoxyuridine; dihydrothymine; malonic acid; methylmalonic acid
28 dpi				
Phenylalanine metabolism	2/45	2.70E−02	0.012	4-Hydroxycinnamic acid; 3-hydroxyphenylacetic acid
Pyrimidine metabolism	2/60	4.59E−02	0.092	Uridine; uracil
Biotin metabolism	1/11	6.23E−02	0.203	Biotin
Caffeine metabolism	1/21	1.16E−01	0.031	Xanthine
Sphingolipid metabolism	1/25	1.36E−01	0	Phytosphingosine
63 dpi				
Valine, leucine, and isoleucine degradation	2/40	2.16E−02	0	l-isoleucine; methylmalonic acid
Glycine, serine, and threonine metabolism	2/48	3.04E−02	0.023	Betaine; l-tryptophan
Aminoacyl-tRNA biosynthesis	2/75	6.85E−02	0	l-isoleucine; l-tryptophan
Purine metabolism	2/92	9.76E−02	0.044	Xanthine; hypoxanthine
Caffeine metabolism	1/21	1.16E−01	0.031	Xanthine

Compound hits represent differential metabolites/all metabolites in a pathway; dpi, days post-infection

### Verification of potential biomarkers

**Fig. 5:**
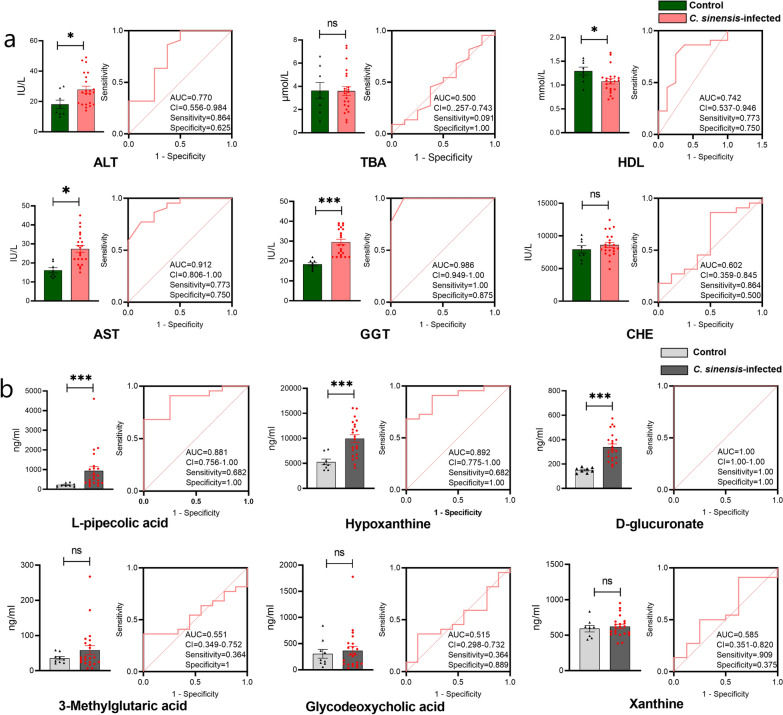
Verification and correlation of biochemical indices and metabolites. **a** The levels of biochemical indices in human serum samples. **b** The contents of candidate diagnostic markers verified via targeted metabolomics. AUC, area under the curve; TBA, total bile acid; CHE, cholinesterase; HDL, high density lipoprotein; ALT, alanine aminotransferase; AST, aspartate transaminase; GGT, glutamyl transpeptidase. ns, non-significant; *P < 0.05, ***P < 0.001 compared to the control group

The baseline characteristics of healthy individuals and patients with *C. sinensis* infection are shown in Table 2. ALT, AST, GGT, and HDL showed diagnostic importance, with GGT and AST showing high AUC values (Fig. 5a). In addition, hypoxanthine, l-pipecolic acid, and d-glucuronate showed significant diagnostic potential (*P* < 0.05 and AUC > 0.8) for human *C. sinensis* infection (Fig. 5b).

**Table 2:** Baseline characteristics of patients and verification indices

Total number of population = 30	Control group	*C. sinensis* group	*P*-value
Sex, *n* (%)			
Male	5 (62.5)	13 (59.1)	
Female	3 (37.5)	9 (40.9)	
Age (years)	45.5 (24–65)	47.0 (36–68)	0.26
ALB (g/l)	43.8 ± 4.6	46.8 ± 4.5	0.12
UA (μmol/l)	268.1 ± 92.4	343.1 ± 152.4	0.20
Harmful alcohol intake*	0	0	
Biochemical indices			
TBA (μmol/l)	3.6 ± 2.0	3.6 ± 1.9	0.96
ALT (IU/l)	18.1 ± 8.0	27.8 ± 10.7	0.03
AST (IU/l)	16.1 ± 3.8	27.4 ± 8.3	0.001
GGT (IU/l)	18.4 ± 2.3	29.6 ± 6.6	< 0.000
HDL (mmol/l)	1.3 ± 0.2	1.1 ± 0.3	0.05
CHE (IU/l)	7945.0 ± 1597.2	8613.1 ± 1804.2	0.36
Metabolites			
l-pipecolic acid (ng/ml)	225.6 ± 30.8	940.3 ± 213.3	0.003
Hypoxanthine (ng/ml)	5279.8 ± 578.6	9963.7 ± 761.0	< 0.000
d-glucuronate (ng/ml)	149.9 ± 7.5	339.3 ± 24.7	< 0.000
3-methylglutaric acid (ng/ml)	32.6 ± 4.1	58.5 ± 12.9	0.07
Glycodeoxycholic acid (ng/ml)	298.9 ± 96.2	363.7 ± 82.3	0.62
Xanthine (ng/ml)	593.3 ± 46.3	622.8 ± 31.3	0.61

Numerical variables are shown as median (range) or mean ± SEM

Categorical variables are expressed *n* (%)

*ALB* albumin, *UA* Uric acid, *TBA* total bile acid, *ALT* alanine aminotransferase, *AST* aspartate transaminase, *GGT* glutamyl transpeptidase, *HDL* high density lipoprotein, *CHE* cholinesterase

*Harmful alcohol intake: > 20 g in women and > 30 g in men

### Correlation analysis

**Fig. 6:**
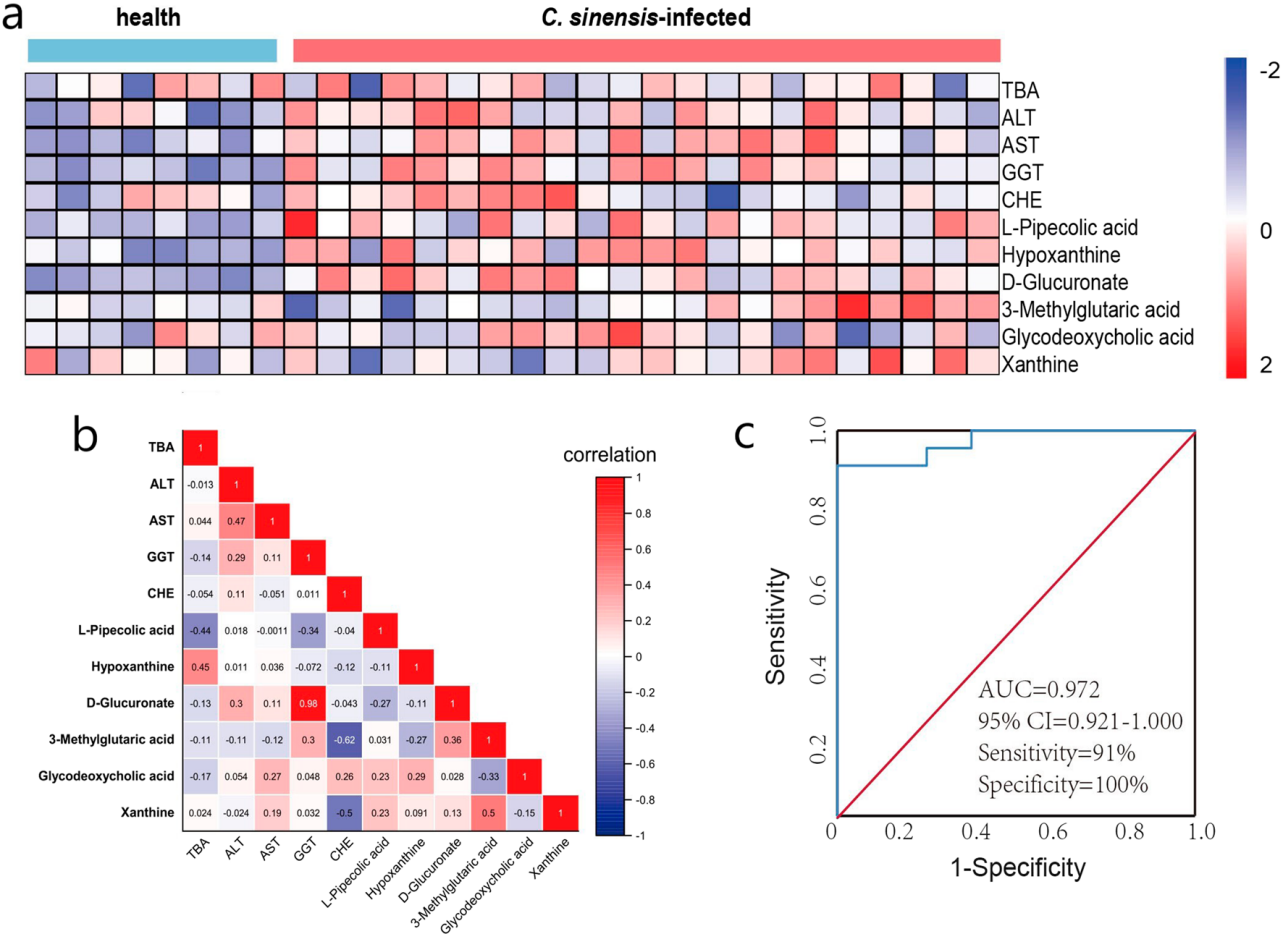
Systematic analysis of key differential metabolites and biochemical indices. **a** Heat map visualization of key differential metabolites and biochemical indices between healthy individuals and *Clonorchis sinensis*-infected patients. **b** Spearman’s correlation was conducted between key differential metabolites and biochemical indices. **c** The receiver-operator characteristic (ROC) curve of gamma-glutamyl transferase (GGT) and d-glucuronate. Positive and negative correlations are indicated using red and blue colors, respectively

The plotted heat map revealed that GGT and d-glucuronate levels were altered in healthy individuals and patients with *C. sinensis* infection, with other candidate biomarkers showing different degrees of alterations (Fig. 6a). GGT level was positively correlated with that of d-glucuronate (*r*(28) = 0.98, *P* < 0.0001) (Fig. 6b). Binary logistic regression analysis of GGT and d-glucuronate for probability prediction and ROC analysis to generate a curve using the obtained probabilities indicated that the panel had an AUC of 0.972, 95% CI: 0.921–1.000 (Fig. 6c). These results demonstrated that this biomarker panel has an excellent diagnostic potential in discriminating patients with *C. sinensis* infection from healthy individuals.

## Discussion

*Clonorchis sinensis* is an important food-borne liver fluke found in certain Asian countries, especially in China [4]. Owing to the absence of apparent symptoms in the early stage of infection, it often leads to a misdiagnosis of clonorchiasis [16]. In this study, analysis of liver tissues from *C. sinensis*-infected rabbits showed a disordered structure of liver lobules, severe hyperplasia of the bile duct, a large area of collagen fiber proliferation, and infiltration of lymphocytes and neutrophils. These symptoms are typical of *C. sinensis* infection and are associated with the development of CCA [9, 17]. In addition, recent studies have shown that the bile acid levels of hosts affect the migration and growth of *C. sinensis* [18], and amino acids levels in hosts were altered after *C. sinensis* infection [19]. Therefore, we speculated that *C. sinensis* would have different effects on the host at different infection stages, and these changes could be observed in the metabolites in plasma, which could serve as biomarkers with diagnostic potential.

The results of biochemical analysis showed that TBA, ALT, AST, and GGT in the serum of rabbits infected with *C. sinensis* were increased, whereas HDL and CHE were decreased. The increase or decrease in ALT, AST, and GGT levels often indicates the occurrence of liver disease. The results of this study were consistent with previous study [9]. Liver is the location of the *C. sinensis* parasite within its hosts, and the effect of its excretion and secretion products on the liver cells has been proved [20]. Therefore, it is reasonable to believe that ALT, AST, and GGT can be used as candidate diagnostic markers for *C. sinensis* infection. In addition, TBA, HDL, and CHE could be considered potential biomarkers. The TBA levels changed especially from 7 to 21 dpi when excysted larvae of *C. sinensis* develop into adults, which indicates that TBA might participate in the growth of *C. sinensis*. Low HDL level up to 9 weeks after infection emphasizes the need for prompt examination of metabolic and inflammatory diseases [21]. In certain parasites, CHE has been reported to play effector roles in host infection [22]. Therefore, these biochemical indices should be considered when evaluating *C. sinensis* infection.

In the present study, using a non-targeted metabolomics-based approach enabled the discovery of plasma metabolites and metabolic pathways associated with the different stages of *C. sinensis* infection. Moreover, it is the first study that explores the plasma metabolic changes using metabolomics. It was found that different stages of the infection were associated with different metabolites, and the most significant changes were observed at 7 and 14 dpi. Previous parasite-associated metabolomic analyses revealed that several metabolites associated with infections may be identified, and that different phases are associated with diverse metabolites [13]. This is because in the early stage of infection the host response to *C. sinensis* is relatively stronger, but as the infection progresses, the response weakens as the host adapts to the foreign substances. Regarding metabolic pathways associated with differential metabolites, we found that 7 and 14 dpi were associated with the largest number of activated pathways. Most of these pathways were related to amino acid metabolism, indicating that amino acid metabolism may play a major part in *C. sinensis* infection. Therefore, it is important to understand the metabolic changes occurring during the infection stage of *C. sinensis*, which may provide information on the development process of CCA. Glutamine and several amino acids were identified in the pathway of central carbon metabolism in cancer. In addition, among the metabolic networks analyzed, seven metabolic pathways were connected, and arginine metabolism covered five metabolic pathways. Several studies have shown that the levels and metabolism of amino acids are closely related to parasitic growth and the development of diseases [19, 23]. Gluconeogenesis and exogenous amino acids may provide energy for the survival of *C. sinensis* in the host [19]. Glutamine, an important nutrient for tumor development, is involved in almost all metabolic functions required for tumor cell proliferation: biosynthesis of macromolecular precursors is involved in bioenergy, antioxidant stress, and glucose metabolism [24]. Although research on glutamine associated with *C. sinensis* infections is limited, based on the studies on other parasites, we speculate that glutamine not only plays an important role in the life cycles of parasites, but also has an inevitable relationship with the occurrence of tumors [25, 26]. In summary, the arginine-glutamine-amino acid metabolic pathways may be associated with the development of clonorchiasis and even CCA.

Biological samples including blood, tissue, urine, cerebrospinal fluid, and feces are commonly used in diagnostic procedures, and plasma is one of the commonly used biological matrices [27]. Therefore, we used human serum samples to verify candidate biomarkers. In this study, the panel of GGT and d-glucuronate had a higher effective diagnostic performance for *C. sinensis* infection. Clinically, GGT level tends to increase in hepatobiliary diseases, which can be used as an auxiliary diagnostic index. d-glucuronate is a carboxylic acid that has the structure of a glucose molecule and is often linked to toxic or poisonous substances. Qian et al. reported that the detection rate of *C. sinensis* using the one-time Kato thick smear method is 11.3%, which is 41.6% lower than the 19.4% infection rate associated with six smear tests according to the gold standard [28]. Li et al. used enzyme-linked immunosorbent assay and found that the specificity of IgG1 and IgG4 was 83.3% and 88.5%, respectively, in patients infected with *C. sinensis* [29]. Through AUC analysis, the panel of GGT and d-glucuronate had a high AUC value (AUC = 0.972), which indicated that the panel of GGT and d-glucuronate has a good diagnostic effect.

We need to address some limitations of this study. First, the sample size for verification was relatively small, which might affect the accuracy of diagnosis; therefore, future studies need verify the diagnosis in large set of samples. Second, other blood sample types from patients with other parasitic infections (*Opisthorchis viverrini*, *Schistosoma mansoni*, fasciolidae parasites, roundworm, and *Echinococcus granulosus*) and with liver diseases (such as HCV or HBV) are required for further analyses, although some blood samples are difficult to obtain.

Taken together, the panel of GGT and d-glucuronate can be used as auxiliary diagnostic indices; meanwhile, it is also necessary to consider the medical history of patients and whether they have visited any *C. sinensis*-endemic areas. In addition, these findings provide new insights to further elucidate the molecular mechanism of clonorchiasis.

## Conclusions

The hosts show a series of metabolic and biochemical changes following infection with *C. sinensis*, and the panel consisting of GGT and d-glucuronate may have good diagnostic potential for *C. sinensis* infection.

## Supplementary Information

**Additional file 1: Figure S1.** Identification of infection and pathological analysis. **Figure S2.** Cluster diagram of biochemical indices at different periods of *Clonorchis sinensis* infection. **Figure S3.** Principal component analysis (PCA) score and unidimensional PCA-X distribution. **Figure S4.** Correlation analysis of the 14 dpi-associated metabolic pathways in central carbon metabolism in cancer.

**Additional file 2: Table S1.** Level of biochemical indices in the *Clonorchis sinensis*-infected group and the control group. **Table S2.** Differential metabolites in *Clonorchis sinensis*-infected rabbits at 7 days post-infection. **Table S3.** Differential metabolites in *Clonorchis sinensis*-infected rabbits at 14 days post-infection. **Table S4.** Differential metabolites in *Clonorchis sinensis*-infected rabbits at 28 days post-infection. **Table S5.** Differential metabolites in *Clonorchis sinensis*-infected rabbits at 63 days post-infection. **Table S6.** Metabolic pathways in different *Clonorchis sinensis* infection phases.

## Data Availability

The datasets supporting the findings of this article are included within the article, and the metabolomics data (MTBLS3306) are available on the MetaboLights database (http://www.ebi.ac.uk/metabolights/).

## Abbreviations

CCA: Cholangiocarcinoma
dpi: Days post-infection
UHPLC-QTOF-MS: Ultra-high-performance liquid chromatography-quadrupole time-of-flight mass spectrometry
ALT: Alanine aminotransferase
AST: Aspartate transaminase
GGT: Glutamyl transpeptidase
TBA: Total bile acid
HDL: High-density lipoprotein
CHE: Cholinesterase
ALP: Alkaline phosphatase
ITS: Internal transcribed spacer
TP: Total protein
ALB: Albumin
GLB: Globulin
LDH: Lactate dehydrogenase
CHOL: Cholesterol
TG: Triglyceride
LDL: Low density lipoprotein
BUN: Blood urea nitrogen
CREA: Creatinine
UA: Uric acid
GLU: Glucose
PA: Prealbumin
QC: Quality control
LC-MS/MS: Liquid chromatography-tandem mass spectrometry
ESI: Electrospray ionization
PCA: Principal component analysis
OPLS-DA: Orthogonal partial least squares discriminant analysis
VIP: Variable importance in projection
ROC: Receiver operating characteristic
AUC: Area under the curve
CI: Confidence interval
