# Supplementary material for: Multiple biochemical indices and metabolomics of Clonorchis sinensis provide a novel interpretation of biomarkers
Source: Parasit Vectors. 2022 May 19;15:172. doi: 10.1186/s13071-022-05290-y (PMC9118806; doi:10.1186/s13071-022-05290-y)
Supplement: Supplementary file 1 — Additional file 1: Figure S1. Identification of infection and pathological analysis. Figure S2. Cluster diagram of biochemical indices at different periods of Clonorchis sinensis infection. Figure S3. Principal component analysis (PCA) score and unidimensional PCA-X distribution. Figure S4. Correlation analysis of the 14 dpi-associated metabolic pathways in central carbon metabolism in cancer. [file 13071_2022_5290_MOESM1_ESM.doc]

Additional file 1

**Multiple biochemical indices and metabolomic of *Clonorchis sinensis* provide a novel interpretation of biomarkers**

**Supporting Information**

**Figure S1.** Identification of infection and pathological analysis.

**Figure S2.** Cluster diagram of biochemical indices at different periods of *Clonorchis sinensis* infection.

**Figure S3.** Principal component analysis (PCA) score and unidimensional PCA-X distribution.

**Figure S4.** Correlation analysis of the 14 days post-infection associated metabolic pathways in central carbon metabolism in cancer.


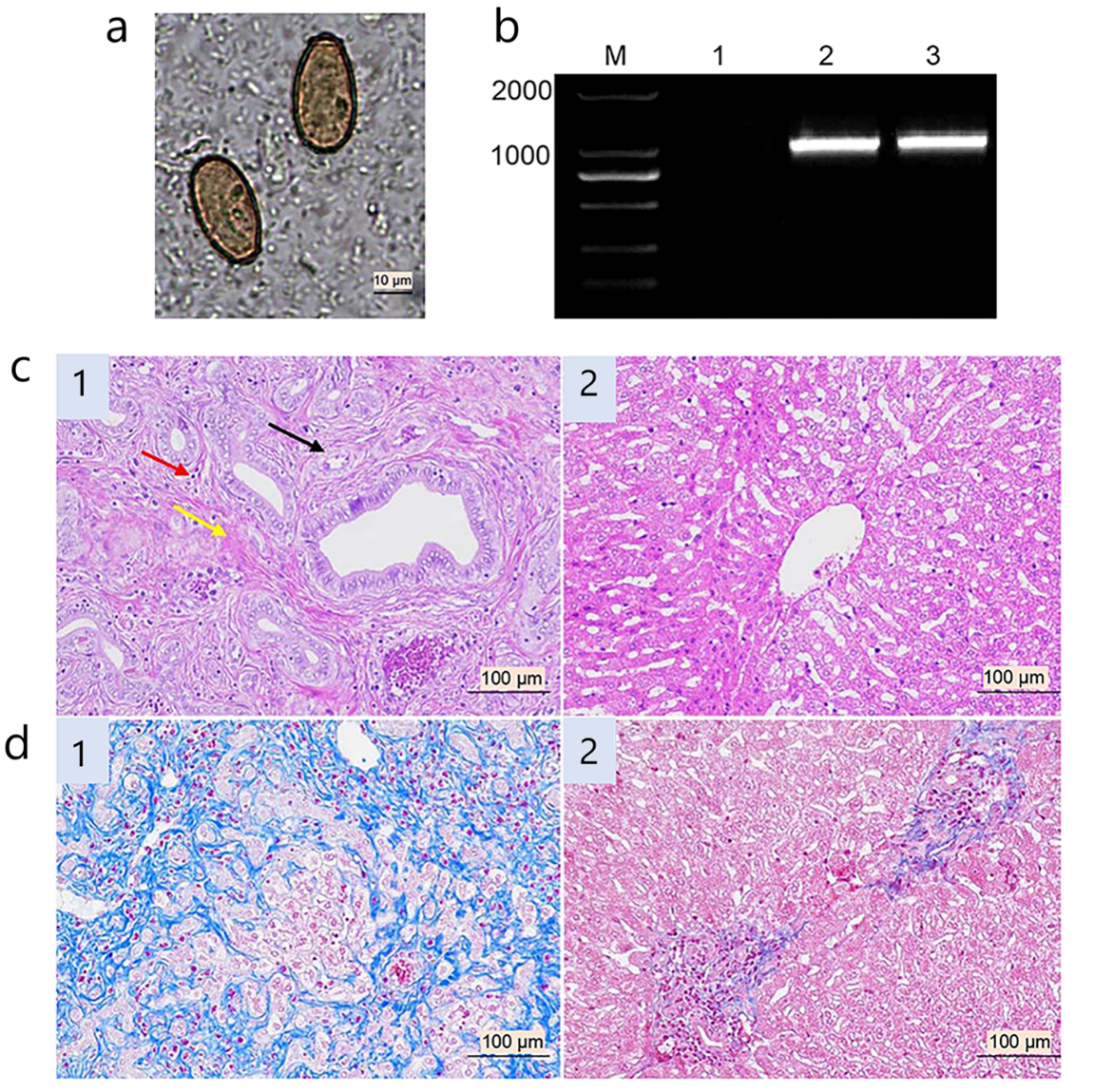


**Figure S1.** **Identification of infection and pathological analysis.**

(a) *Clonorchis sinensis* eggs observed under an optical microscope. (b) Internal transcribed spacers (ITS) identification of *C. sinensis* eggs in stool. M is the DL2000 DNA Marker; lanes 1–3 represent negative control, *C. sinensis* stool 1 and 2. (c and d) Hematoxylin and eosin (H&E) and Masson’s trichrome staining for *C. sinensis*-infected (1) and control (2) rabbit liver. Black arrow, bile duct; yellow arrow, area of collagen fiber hyperplasia; red arrow, increased levels of lymphocytes and neutrophils.

**
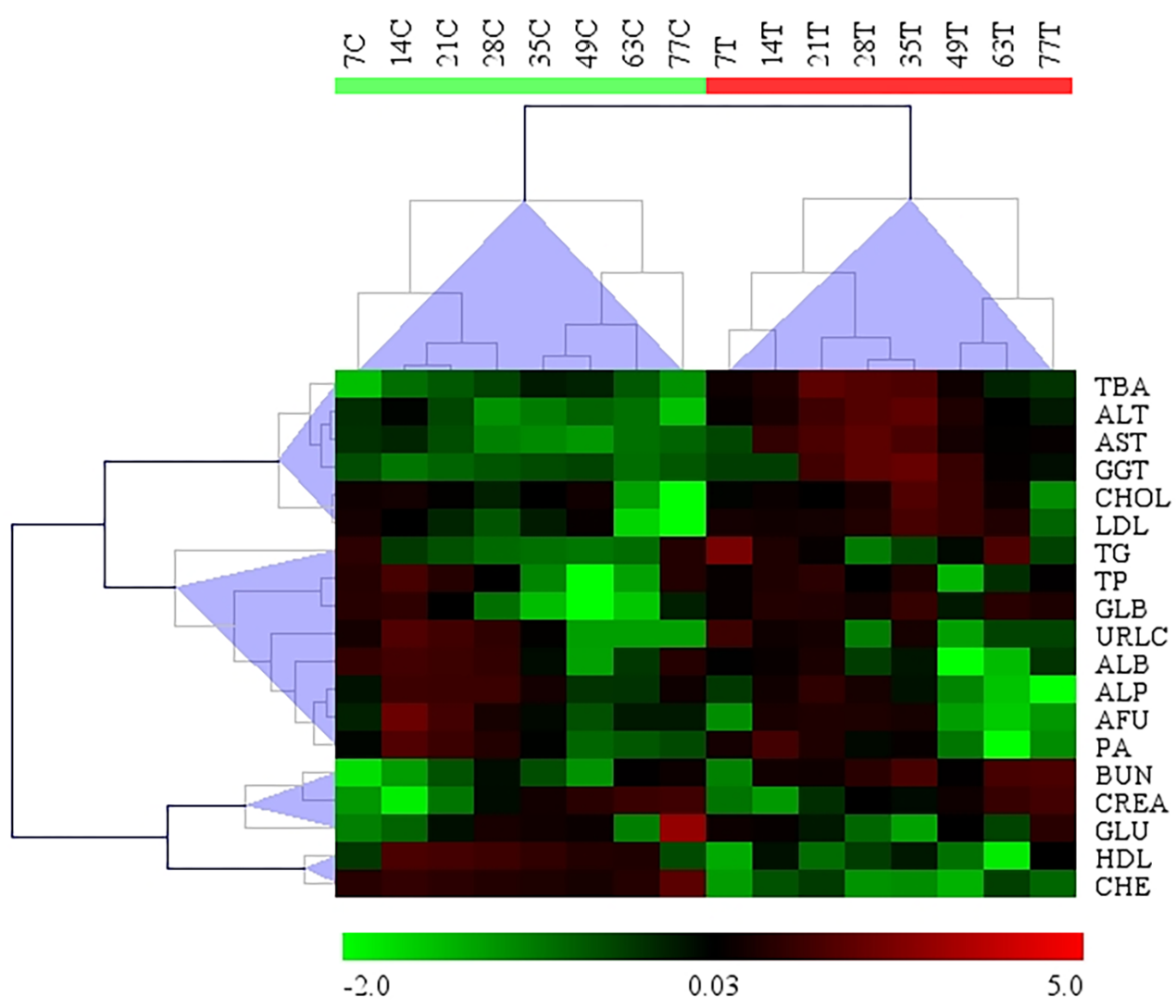
**

**Figure S2.** Cluster diagram of biochemical indices at different periods of *Clonorchis sinensis* infection. dpi, days post-infection; C, control group; T, *C. sinensis*-infected group.

**
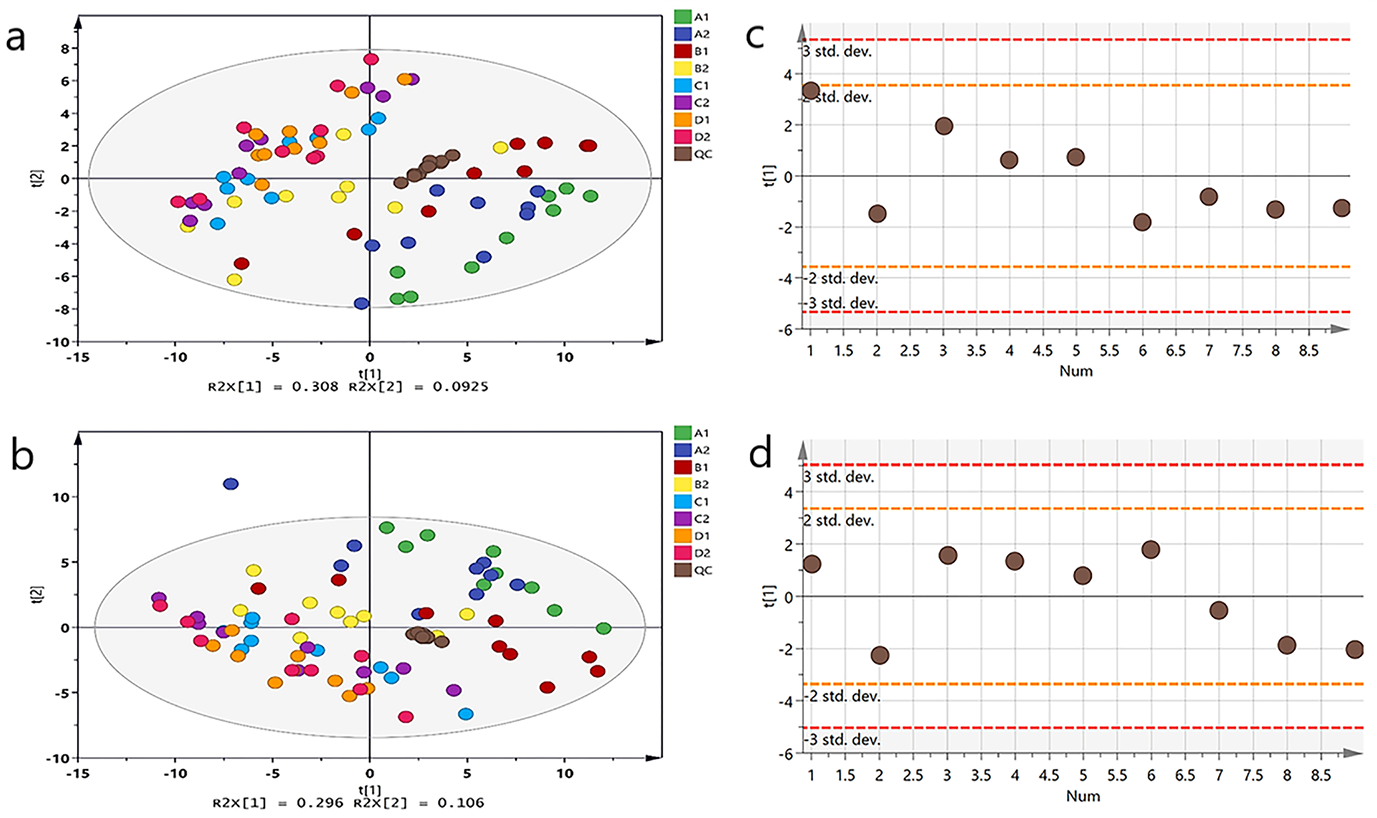
**

**Figure S3.** Principal component analysis (PCA) score of all samples (including QC samples) and unidimensional PCA-X distribution of QC samples. (a) PCA score in positive electrospray ionization (ESI+) mode. (b) PCA score in negative electrospray ionization (ESI−). (c) Unidimensional PCA-X distribution of QC samples in ESI+ mode. (d) Unidimensional PCA-X distribution of QC samples in ESI− mode. 1, control group; 2, *C. sinensis*-infected group; A, 7 days post-infection (dpi); B, 14 dpi; C, 28 dpi; D, 63 dpi.

**
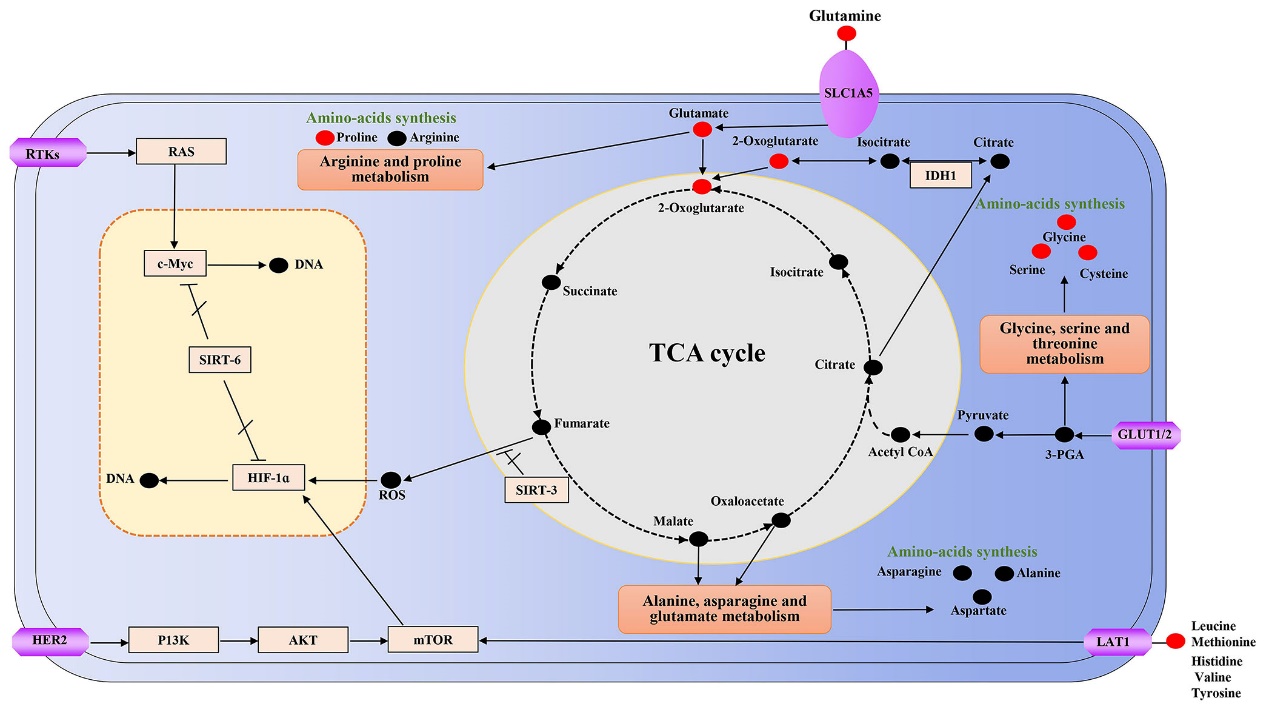
**

**Figure S4.** Correlation analysis of the 14 dpi-associated metabolic pathways in central carbon metabolism in cancer. Oncogenes (Ras, PI3K, Akt, and c-Myc) and tumor suppressor genes (SIRT3 and SIRT6); the red circles represent the differential metabolites, and the black circles represent the original metabolites in the pathway. dpi, days post-infection; PI3K, phosphoinositide 3-kinase; Akt, protein kinase B; SIRT3, silent mating type information regulation 2 homolog 3.
