# Supplementary material for: Multiple biochemical indices and metabolomics of Clonorchis sinensis provide a novel interpretation of biomarkers
Source: Parasit Vectors. 2022 May 19;15:172. doi: 10.1186/s13071-022-05290-y (PMC9118806; doi:10.1186/s13071-022-05290-y)
Supplement: Supplementary file 2 — Additional file 2: Table S1. Level of biochemical indices in the Clonorchis sinensis-infected group and the control group. Table S2. Differential metabolites in Clonorchis sinensis-infected rabbits at 7 days post-infection. Table S3. Differential metabolites in Clonorchis sinensis-infected rabbits at 14 days post-infection. Table S4. Differential metabolites in Clonorchis sinensis-infected rabbits at 28 days post-infection. Table S5. Differential metabolites in Clonorchis sinensis-infected rabbits at 63 days post-infection. Table S6. Metabolic pathways in different Clonorchis sinensis infection phases. [file 13071_2022_5290_MOESM2_ESM.doc]

Additional file 2

**Multiple biochemical indices and metabolomic of *Clonorchis sinensis* provide a novel interpretation of biomarkers**

**Supporting Information**

**Table S1.** Level of biochemical indices in the *Clonorchis sinensis*-infected group and the control group.

**Table S2.** Differential metabolites in *Clonorchis sinensis*-infected rabbits at 7 days post-infection.

**Table S3.** Differential metabolites in *Clonorchis sinensis*-infected rabbits at 14 days post-infection.

**Table S4.** Differential metabolites in *Clonorchis sinensis*-infected rabbits at 28 days post-infection.

**Table S5.** Differential metabolites in *Clonorchis sinensis*-infected rabbits at 63 days post-infection.

**Table S6.** Metabolic pathways in different *Clonorchis sinensis* infection phases.

**Table S1. Level of biochemical indices in the *Clonorchis sinensis-*infected group and the control group (mean ± SEM).**

|  | 7 dpi | | 14 dpi | | 21 dpi | | 28 dpi | |
| --- | --- | --- | --- | --- | --- | --- | --- | --- |
|  | C | T | C | T | C | T | C | T |
| TP | 71.92±1.25 | 69.89±1.35 | 74.34±2.75 | 71.55±1.91 | 71.93±2.26 | 72.33±1.80 | 69.53±1.52 | 69.41±1.81 |
| ALB | 42.66±0.60 | 40.27±1.00 | 43.35±1.12 | 40.71±0.47* | 43.01±0.94 | 41.59±0.59 | 42.67±0.58 | 39.25±0.60** |
| GLB | 31.01±0.96 | 29.62±1.02 | 31.44±1.79 | 30.84±2.02 | 29.35±1.28 | 30.74±1.47 | 27.25±0.92 | 30.17±1.49 |
| TBA | 17.46±2.28 | 31.90±1.59*** | 21.58±0.73 | 35.62±4.80* | 22.86±2.46 | 52.93±7.97*** | 24.18±0.86 | 49.82±5.69*** |
| ALT | 57.56±1.38 | 66.78±4.62 | 63.44±0.82 | 74.22±5.03 | 54.56±1.99 | 92.00±6.72*** | 45.56±3.62 | 103.00±6.88*** |
| AST | 28.22±1.88 | 26.00±1.31 | 29.56±2.04 | 54.44±9.32* | 25.33±1.17 | 69.44±9.43*** | 20.78±1.01 | 82.00±11.62*** |
| GGT | 9.56±0.69 | 10.22±0.60 | 8.11±0.86 | 10.22±1.60 | 8.89±0.61 | 25.22±5.82* | 9.22±0.46 | 33.00±5.99** |
| LDH | 373.01±52.29 | 84.43±5.22*** | 175.98±10.25 | 118.82±17.37* | 164.53±6.93 | 99.97±12.42*** | 153.03±6.10 | 102.74±7.21*** |
| CHOL | 2.98±0.21 | 2.74±0.25 | 3.05±0.04 | 2.92±0.11 | 2.81±0.09 | 2.76±0.28 | 2.58±0.14 | 3.12±0.22 |
| TG | 0.95±0.06 | 1.48±0.10*** | 0.63±0.01 | 0.87±0.13 | 0.60±0.03 | 0.75±0.08 | 0.56±0.05 | 0.54±0.02 |
| HDL | 1.27±0.09 | 0.73±0.08*** | 1.44±0.06 | 0.98±0.09*** | 1.40±0.02 | 0.79±0.03*** | 1.34±0.05 | 0.90±0.03*** |
| LDL | 1.60±0.12 | 1.60±0.21 | 1.34±0.02 | 1.54±0.10 | 1.14±0.04 | 1.60±0.23 | 0.94±0.07 | 1.81±0.13*** |
| BUN | 6.29±0.04 | 6.81±0.62 | 6.65±0.16 | 7.91±0.43* | 7.02±0.08 | 7.89±0.43 | 7.55±0.08 | 8.17±0.35 |
| CREA | 78.26±2.97 | 81.20±1.64 | 71.47±0.70 | 78.03±3.49 | 81.26±0.90 | 87.24±2.80 | 90.60±1.40 | 91.98±2.31 |
| UA | 3.22±0.70 | 4.56±0.60 | 5.56±1.07 | 3.00±0.55 | 4.89±1.09 | 3.22±0.91 | 4.00±1.17 | 1.56±0.24 |
| GLU | 6.78±0.09 | 7.83±0.16*** | 6.95±0.24 | 7.74±0.20* | 7.50±0.29 | 7.44±0.33 | 8.03±0.35 | 6.93±0.15* |
| PA | 287.76±5.89 | 306.12±13.70 | 357.03±4.70 | 344.61±12.48 | 337.44±1.74 | 314.88±16.60 | 317.84±5.22 | 287.04±15.40 |
| CHE | 3517.00±311.15 | 2846.17±148.32 | 3623.50±359.63 | 2888.80±36.01 | 3556.00±341.62 | 2976.00±129.42 | 3488.25±353.45 | 2706.60±33.78 |

|  | 35 dpi | | 49 dpi | | 63 dpi | | 77 dpi | |
| --- | --- | --- | --- | --- | --- | --- | --- | --- |
|  | C | T | C | T | C | T | C | T |
| TP | 65.85±0.90 | 71.62±2.71 | 62.17±0.54 | 64.62±3.50 | 65.12±1.00 | 68.18±1.04 | 71.87±0.71 | 69.62±0.94 |
| ALB | 40.11±0.41 | 39.92±0.84 | 37.52±0.47 | 35.86±1.54 | 39.31±0.54 | 37.07±0.59* | 41.97±0.70 | 39.39±0.55* |
| GLB | 25.95±0.74 | 31.70±1.73** | 24.65±0.48 | 28.77±2.23 | 25.80±0.67 | 31.10±1.23** | 28.67±0.77 | 30.58±0.68 |
| TBA | 26.93±0.95 | 47.50±1.67*** | 26.34±0.78 | 31.90±1.20** | 22.86±1.00 | 26.36±1.51 | 19.70±1.34 | 25.24±2.71 |
| ALT | 48.22±2.53 | 111.00±11.08*** | 50.78±1.66 | 76.56±5.03*** | 49.22±2.59 | 64.44±7.05 | 40.78±2.81 | 60.44±3.70** |
| AST | 20.11±0.65 | 67.22±8.29*** | 19.00±0.76 | 41.44±4.85*** | 22.00±1.26 | 35.22±2.24*** | 23.00±1.04 | 36.22±2.60*** |
| GGT | 9.78±0.28 | 36.22±5.00*** | 10.00±0.55 | 22.78±3.75** | 8.44±0.47 | 13.78±1.02*** | 9.22±0.43 | 12.44±0.56*** |
| LDH | 102.96±3.94 | 107.51±13.22 | 52.84±3.12 | 70.79±3.42** | 91.46±6.20 | 75.93±8.77 | 130.50±13.48 | 167.13±13.86 |
| CHOL | 2.79±0.10 | 4.13±0.27*** | 2.88±0.11 | 3.68±0.38 | 1.98±0.05 | 2.95±0.74 | 1.38±0.13 | 2.08±0.03*** |
| TG | 0.55±0.04 | 0.62±0.02 | 0.54±0.04 | 0.70±0.07 | 0.56±0.02 | 0.50±0.08 | 0.90±0.04 | 0.62±0.03*** |
| HDL | 1.27±0.05 | 0.97±0.02*** | 1.20±0.06 | 0.82±0.04*** | 1.17±0.03 | 0.65±0.05*** | 0.88±0.08 | 0.86±0.06 |
| LDL | 1.17±0.05 | 2.60±0.11*** | 1.40±0.05 | 2.30±0.22** | 0.57±0.02 | 0.79±0.11 | 0.39±0.06 | 0.88±0.06*** |
| BUN | 7.13±0.06 | 8.87±0.32*** | 6.72±0.11 | 7.67±0.33* | 7.68±0.15 | 8.54±1.09 | 9.85±0.18 | 8.97±0.17** |
| CREA | 96.32±2.21 | 90.72±2.26 | 102.07±2.91 | 95.36±4.38 | 106.50±2.26 | 105.68±2.60 | 117.84±0.74 | 109.10±4.63 |
| UA | 2.78±0.52 | 3.22±0.52 | 1.44±0.18 | 1.44±0.18 | 1.44±0.18 | 2.00±0.55 | 9.44±1.67 | 15.56±1.25* |
| GLU | 7.91±0.15 | 6.57±0.23*** | 7.78±0.10 | 7.61±0.40 | 6.78±0.13 | 7.15±0.16 | 10.50±0.38 | 8.33±0.21*** |
| PA | 288.82±6.98 | 296.04±12.95 | 259.81±10.29 | 255.50±20.39 | 263.82±3.26 | 217.04±23.32 | 267.88±9.75 | 249.68±3.19 |
| CHE | 3386.88±246.94 | 2723.40±113.48 | 3369.88±247.28 | 2620.00±107.31 | 3496.29±270.69 | 2960.00±168.65 | 3669.88±237.81 | 2841.00±40.70* |

Note: The difference between the *C. sinensis*-infected and the control group at each time point of each indice was obtained by two-way ANOVA analysis. Different letters marked differences between the two groups (**P* < 0.05, ***P* < 0.01, ****P* < 0.001).

C, control group; T, *C. sinensis*-infected group; dpi, days post-infection.

TP, total protein; ALB, albumin; GLB, globulin; TBA, total bile acid; ALT, alanine aminotransferase; AST, aspartate transaminase; GGT, glutamyl transpeptidase; LDH, lactate dehydrogenase; CHOL, cholesterol; TG, triglyceride; HDL, high density lipoprotein; LDL, low density lipoprotein; BUN, blood urea nitrogen; CREA, creatinine; UA, uric acid; GLU, glucose; PA, prealbumin; CHE, cholinesterase.

**Table S2.** Differential metabolites in *Clonorchis sinensis*-infected rabbit at 7 days post-infection.

| ID | MS2 name | m/z | VIP | *P*-Value | Fold change | ESI |
| --- | --- | --- | --- | --- | --- | --- |
| 1 | Ubiquinone (Q2) | 363.1559918 | 2.334000353 | 0.013321453 | 0.45909461 | ESI+ |
| 2 | *S*-Methyl-5'-thioadenosine | 298.0935804 | 1.514887606 | 0.023829776 | 1.638110763 | ESI+ |
| 3 | *N*-Docosanoyl-4-sphingenyl-1-O-phosphorylcholine | 825.6170611 | 1.619750154 | 0.037243277 | 0.65635379 | ESI+ |
| 4 | *N*-(omega)-Hydroxyarginine | 232.1373873 | 2.038733365 | 0.037429699 | 0.557094117 | ESI+ |
| 5 | Ile-Lys | 557.3476817 | 2.742458677 | 0.017469696 | 3.399074727 | ESI+ |
| 6 | Dopamine | 324.1837607 | 2.202187411 | 0.025765767 | 2.44651666 | ESI+ |
| 7 | Diethyltoluamide | 192.1354993 | 1.112543714 | 0.026211847 | 0.65321969 | ESI+ |
| 8 | Cytosine | 112.0486335 | 1.809570111 | 0.034356829 | 0.795269176 | ESI+ |
| 9 | Ala-Leu | 203.1391078 | 2.151711271 | 0.023659355 | 2.709018159 | ESI+ |
| 10 | 3-Methylindole | 132.0779986 | 1.901418669 | 0.024691044 | 1.939358568 | ESI+ |
| 11 | 3-Methylglutaric acid | 188.088954 | 1.799460837 | 0.012697609 | 0.637896406 | ESI+ |
| 12 | γ-l-Glu-ε.-l-Lys | 336.1734024 | 2.075655294 | 0.028193824 | 1.477026623 | ESI+ |
| 13 | Uracil | 111.020074 | 1.888450988 | 0.001186563 | 0.545859176 | ESI− |
| 14 | Trans-cinnamate | 148.0505382 | 1.466550716 | 0.029586922 | 0.66147324 | ESI− |
| 15 | Thymidine | 241.0822337 | 1.39941627 | 0.022410477 | 0.693879325 | ESI− |
| 16 | Sphingosine-1-phosphate | 378.2394404 | 1.456110495 | 0.026015155 | 0.698027165 | ESI− |
| 17 | Sarcosine | 88.03981469 | 1.687611742 | 0.008354443 | 0.635941913 | ESI− |
| 18 | Rosolic acid | 289.0929426 | 1.784758032 | 0.017234157 | 2.526022834 | ESI− |
| 19 | Ribothymidine | 279.054457 | 1.61437632 | 0.013505212 | 0.740270861 | ESI− |
| 20 | Pyruvaldehyde | 131.036863 | 1.775714645 | 0.012682549 | 0.568581815 | ESI− |
| 21 | Pioglitazone | 337.1097321 | 1.634218947 | 0.040214748 | 0.474441359 | ESI− |
| 22 | p-Hydroxycinnamaldehyde | 185.0011304 | 2.047515353 | 0.003773025 | 0.440525646 | ESI− |
| 23 | Phenylacetylglycine | 192.0662824 | 1.675320851 | 0.017780626 | 0.421852935 | ESI− |
| 24 | Perseitol | 193.070691 | 1.863347736 | 0.009052643 | 0.64456497 | ESI− |
| 25 | Pectin (galacturonic acid) | 193.0341871 | 1.407838567 | 0.047192243 | 0.751772385 | ESI− |
| 26 | Oxypurinol | 133.0145843 | 1.869166069 | 0.01730995 | 0.58576921 | ESI− |
| 27 | *O*-Phospho-l-threonine | 199.0213922 | 1.389243801 | 0.049869141 | 0.484607802 | ESI− |
| 28 | Nicotinamide ribotide | 333.0571167 | 1.427588796 | 0.036179875 | 0.592605489 | ESI− |
| 29 | *N*-Acetylneuraminic acid | 309.1002023 | 1.369331752 | 0.025625942 | 0.711016462 | ESI− |
| 30 | *N*-Acetylmannosamine | 220.0816794 | 1.550007078 | 0.03781903 | 0.686920035 | ESI− |
| 31 | *N*-Acetyl-l-phenylalanine | 206.0813136 | 1.822428933 | 0.015717618 | 0.606309291 | ESI− |
| 32 | *N*2-Acetyl-l-ornithine | 173.0920188 | 1.625270758 | 0.02526609 | 0.694701246 | ESI− |
| 33 | Monomethyl glutaric acid | 162.0760105 | 1.598749905 | 0.030356407 | 0.712467395 | ESI− |
| 34 | Methylmalonic acid | 117.0185495 | 1.559614279 | 0.044321605 | 0.581567638 | ESI− |
| 35 | Maleamic acid | 174.0401038 | 1.512114298 | 0.037942382 | 0.653196587 | ESI− |
| 36 | l-Proline | 114.055766 | 1.269978331 | 0.040644738 | 0.734126396 | ESI− |
| 37 | l-Homocysteic acid | 199.0366447 | 1.473590763 | 0.044090201 | 0.699617355 | ESI− |
| 38 | l-Histidine | 154.0614937 | 1.533521533 | 0.016491152 | 0.670006504 | ESI− |
| 39 | l-Glutamine | 145.0616489 | 1.405319032 | 0.029495027 | 0.824658332 | ESI− |
| 40 | l-Asparagine | 131.045605 | 1.43180826 | 0.031734805 | 0.752810541 | ESI− |
| 41 | Indole-3-carboxylic acid | 160.0394329 | 1.164036174 | 0.025275364 | 0.619820763 | ESI− |
| 42 | Hydroxyisocaproic acid | 131.0705556 | 1.639054268 | 0.01511805 | 0.704431306 | ESI− |
| 43 | Glycine | 74.02469357 | 1.262188436 | 0.035345279 | 0.735352035 | ESI− |
| 44 | d-Ornithine | 131.0820674 | 1.374796275 | 0.028696302 | 0.780854193 | ESI− |
| 45 | dl-Lactate | 89.02452651 | 1.472953975 | 0.035392809 | 0.673769529 | ESI− |
| 46 | Dihydroxyfumarate | 147.0654054 | 1.371376443 | 0.032095323 | 0.653299653 | ESI− |
| 47 | Deoxycytidine | 286.1027254 | 1.525059763 | 0.019530711 | 0.625014578 | ESI− |
| 48 | d(-)-beta-hydroxy butyric acid | 163.0602347 | 1.762180649 | 0.008296443 | 0.626214729 | ESI− |
| 49 | Cytidine | 242.0774919 | 1.627183278 | 0.021323623 | 0.762253765 | ESI− |
| 50 | Citramalic acid | 207.0497378 | 1.335234682 | 0.040908592 | 0.425615959 | ESI− |
| 51 | Ammelide | 128.0346964 | 1.477701576 | 0.019771666 | 0.807170242 | ESI− |
| 52 | Alpha-ketoisovaleric acid | 115.0396748 | 1.845753206 | 0.006102383 | 0.465220024 | ESI− |
| 53 | 3-Hexanone | 121.0650761 | 1.50371017 | 0.036361178 | 2.340329108 | ESI− |
| 54 | 3-Aminopropanesulphonic Acid | 277.058363 | 1.531166789 | 0.019683845 | 0.680995348 | ESI− |
| 55 | 2-Oxoadipic acid | 141.0166001 | 1.383980975 | 0.042779643 | 0.795044311 | ESI− |
| 56 | 2'-Deoxyuridine | 227.0667242 | 1.556391967 | 0.02639021 | 0.571005671 | ESI− |
| 57 | 2'-Deoxy-D-ribose | 267.1086871 | 1.601248059 | 0.035599085 | 0.629687838 | ESI− |
| 58 | 1-Stearoyl-sn-glycerol 3-phosphocholine | 522.3538523 | 1.546275598 | 0.033657368 | 0.773200684 | ESI− |

MS2, secondary mass spectrometry; m/z, mass-to-charge ratio; VIP, variable importance in projection; ESI, electrospray ionization.

**Table S3.** Differential metabolites in *Clonorchis sinensis*-infected rabbit at 14 days post-infection.

| ID | MS2 name | m/z | VIP | *P*-Value | Fold change | ESI |
| --- | --- | --- | --- | --- | --- | --- |
| 1 | Xanthosine | 285.082879 | 1.84572236 | 2.37523E-05 | 0.529409565 | ESI+ |
| 2 | Xanthine | 153.0380672 | 1.727324351 | 0.000801202 | 0.271283993 | ESI+ |
| 3 | Val-Leu | 248.2024755 | 1.15246613 | 0.038939043 | 0.675415689 | ESI+ |
| 4 | Valeric acid | 144.0990286 | 1.240663793 | 0.024634559 | 0.705578735 | ESI+ |
| 5 | Val-Arg | 274.1924919 | 1.523141157 | 0.010448919 | 0.516069844 | ESI+ |
| 6 | Ubiquinone (Q2) | 363.1559918 | 1.60573012 | 0.006307973 | 0.316269827 | ESI+ |
| 7 | Tyr-Glu | 371.1417482 | 1.072661667 | 0.014112055 | 0.462218366 | ESI+ |
| 8 | Trans-2-Hydroxycinnamic acid | 165.0520391 | 1.525750385 | 0.002068282 | 0.744291094 | ESI+ |
| 9 | Thr-Tyr | 346.1344893 | 1.150657401 | 0.015611773 | 0.496035769 | ESI+ |
| 10 | Thr-Glu | 249.1130525 | 1.187531594 | 0.022719911 | 0.339271049 | ESI+ |
| 11 | Taurolithocholic acid | 484.3009514 | 1.404874239 | 0.022091062 | 0.533230681 | ESI+ |
| 12 | Stearic acid | 302.3029671 | 1.629696683 | 0.014285129 | 0.356059456 | ESI+ |
| 13 | sn-Glycerol 3-phosphoethanolamine | 216.0603297 | 1.485431109 | 0.019139402 | 0.612820417 | ESI+ |
| 14 | Quinaldic acid | 174.0523831 | 1.041922572 | 0.015792001 | 0.661130002 | ESI+ |
| 15 | Pyruvaldehyde | 114.0525263 | 1.411075367 | 0.038225843 | 0.760153462 | ESI+ |
| 16 | Prunasin | 295.1113064 | 1.217456377 | 0.012371355 | 0.354744193 | ESI+ |
| 17 | Pro-Thr | 277.1367672 | 1.098861266 | 0.036329016 | 0.513727807 | ESI+ |
| 18 | Pro-Glu | 289.0747628 | 1.342538726 | 0.006284509 | 0.475171278 | ESI+ |
| 19 | Pro-Arg | 271.1626395 | 1.235244928 | 0.04880219 | 0.582653849 | ESI+ |
| 20 | Phenylpropionylglycine | 268.1236215 | 1.466203538 | 0.011520662 | 0.548894002 | ESI+ |
| 21 | Phenylacetic acid | 119.0507137 | 1.090597932 | 0.007990238 | 0.422322052 | ESI+ |
| 22 | Phenacetine | 243.1060024 | 1.352646656 | 0.013175035 | 0.351288484 | ESI+ |
| 23 | Pentadecanoic Acid | 260.2560375 | 1.640942708 | 0.00199164 | 0.312267062 | ESI+ |
| 24 | p-Chlorophenylalanine | 200.0446596 | 1.311355415 | 0.026876273 | 0.476946552 | ESI+ |
| 25 | PC(20:5(5Z,8Z,11Z,14Z,17Z)/20:5(5Z,8Z,11Z,14Z,17Z)) | 890.5505355 | 1.155026822 | 0.024959031 | 0.523725908 | ESI+ |
| 26 | Pargyline | 182.0912577 | 1.608012944 | 0.003935037 | 0.488166039 | ESI+ |
| 27 | Palmitic acid | 274.271844 | 1.637567324 | 0.003493212 | 0.378150217 | ESI+ |
| 28 | Oxprenolol | 531.3378354 | 1.139075592 | 0.012433683 | 0.706190204 | ESI+ |
| 29 | Oxaprozin | 294.1158493 | 1.191313579 | 0.016122139 | 0.264899067 | ESI+ |
| 30 | *O*-Acetyl-l-serine | 295.1199104 | 1.483937667 | 0.009360891 | 0.296542004 | ESI+ |
| 31 | Norharmane | 359.125232 | 1.427569176 | 0.007814656 | 0.457434404 | ESI+ |
| 32 | NG,NG-dimethyl-l-arginine (ADMA) | 203.1481383 | 1.578040648 | 0.004283635 | 0.71994915 | ESI+ |
| 33 | *N*-Acetyl-l-glutamic acid | 231.0950253 | 1.614479744 | 0.008060491 | 0.595070065 | ESI+ |
| 34 | N6-Methyl-l-lysine | 161.1256448 | 1.607708682 | 0.02845992 | 0.691548364 | ESI+ |
| 35 | *N*-(omega)-Hydroxyarginine | 232.1373873 | 1.647483811 | 0.007506265 | 0.447308861 | ESI+ |
| 36 | Myristic acid | 246.2402275 | 1.632023131 | 0.002141401 | 0.30553568 | ESI+ |
| 37 | Met-Tyr | 312.1096695 | 1.222043815 | 0.043761218 | 0.488708358 | ESI+ |
| 38 | Methylmalonic acid | 119.0327104 | 1.651301585 | 0.000159373 | 0.342302346 | ESI+ |
| 39 | l-Tyrosine | 182.0787152 | 1.477843853 | 0.001551714 | 0.749521317 | ESI+ |
| 40 | l-Threonine | 120.0629873 | 1.114601185 | 0.004098349 | 0.393702526 | ESI+ |
| 41 | l-Serine | 106.0474478 | 1.006877715 | 0.018055202 | 0.501897997 | ESI+ |
| 42 | l-Proline | 116.0688373 | 1.666876723 | 0.001107476 | 0.665450197 | ESI+ |
| 43 | Lomefloxacin | 352.1475128 | 1.11086953 | 0.040894785 | 0.446354633 | ESI+ |
| 44 | l-NG-Monomethylarginine | 189.132027 | 1.409084726 | 0.013512395 | 0.733471446 | ESI+ |
| 45 | l-Methionine | 150.0555745 | 1.355685128 | 0.018981732 | 0.737750513 | ESI+ |
| 46 | l-Lysine | 146.1043468 | 1.180513981 | 0.019480445 | 0.648457537 | ESI+ |
| 47 | l-Leucine | 132.0995211 | 1.358127749 | 0.011395722 | 0.756573461 | ESI+ |
| 48 | Linoleic acid | 303.2290845 | 1.425414049 | 0.031968204 | 0.436144236 | ESI+ |
| 49 | l-Histidine | 156.0741695 | 1.309795643 | 0.009507337 | 0.712577417 | ESI+ |
| 50 | l-Glutamine | 147.0737722 | 1.143490754 | 0.020566818 | 0.802886607 | ESI+ |
| 51 | l-Glutamate | 148.0579198 | 1.457498303 | 0.012356702 | 0.572259601 | ESI+ |
| 52 | l-Cysteine | 243.0409714 | 1.54300309 | 0.002445478 | 0.501691842 | ESI+ |
| 53 | l-Citrulline | 176.1006387 | 1.452814737 | 0.007750375 | 0.761666882 | ESI+ |
| 54 | l-Carnitine | 162.11048 | 1.192483273 | 0.030730059 | 0.71530403 | ESI+ |
| 55 | Isovalerylglycine | 220.1153967 | 1.528562294 | 0.008403762 | 0.561173566 | ESI+ |
| 56 | Inosine | 269.0855987 | 1.22550279 | 0.016599196 | 0.448399078 | ESI+ |
| 57 | Indole-2-carboxylic acid | 162.0546874 | 1.485169392 | 0.045796967 | 0.48015297 | ESI+ |
| 58 | Ile-Thr | 482.324177 | 1.069710719 | 0.020651141 | 0.675739499 | ESI+ |
| 59 | Ile-Cys | 276.1416701 | 1.250013962 | 0.027938095 | 0.607313484 | ESI+ |
| 60 | Hypoxanthine | 137.0436365 | 1.208522665 | 0.02579649 | 0.438816372 | ESI+ |
| 61 | Hydroxyacetone | 116.068391 | 1.389055631 | 0.005081523 | 0.70684217 | ESI+ |
| 62 | His-Pro | 235.1145656 | 1.448558438 | 0.038713807 | 0.619092251 | ESI+ |
| 63 | Hippuric acid | 180.0627382 | 1.398406846 | 0.029548418 | 0.644448428 | ESI+ |
| 64 | Hexanoic acid | 116.0815939 | 1.280824649 | 0.020863984 | 0.756206886 | ESI+ |
| 65 | Heptadecanoic acid | 288.2872094 | 1.234521049 | 0.006181983 | 0.514374434 | ESI+ |
| 66 | Guanosine | 284.1044684 | 1.528525315 | 0.004675541 | 0.667593278 | ESI+ |
| 67 | Guanidineacetic acid | 162.0220788 | 1.625355096 | 0.003505926 | 0.579867766 | ESI+ |
| 68 | Glycylproline | 173.0890056 | 1.539326617 | 0.026571035 | 0.443920085 | ESI+ |
| 69 | Glycerophosphocholine | 258.1082034 | 1.17962707 | 0.037509451 | 0.695666234 | ESI+ |
| 70 | Glutaraldehyde | 223.0902129 | 1.064914744 | 0.017875525 | 0.327130117 | ESI+ |
| 71 | Fastigilin B | 385.1615083 | 1.513957647 | 0.013676197 | 0.510405267 | ESI+ |
| 72 | Famciclovir | 366.1129099 | 1.012794498 | 0.015766039 | 0.346451958 | ESI+ |
| 73 | Ethylmalonic acid | 282.1155347 | 1.19751133 | 0.013681439 | 0.359353031 | ESI+ |
| 74 | Ethyl hydrogen malonate | 196.0576586 | 1.090549844 | 0.042555058 | 0.591018021 | ESI+ |
| 75 | d-Ornithine | 196.1053702 | 1.50292303 | 0.011643269 | 0.308425815 | ESI+ |
| 76 | d-Mannose | 145.0470199 | 1.119692991 | 0.037477623 | 0.724977785 | ESI+ |
| 77 | dl-Vanillylmandelic acid | 216.0840178 | 1.142636418 | 0.023936019 | 0.473137691 | ESI+ |
| 78 | dl-Homoserine | 180.0837823 | 1.074009663 | 0.016786951 | 0.589998728 | ESI+ |
| 79 | Dimethylglycine | 104.0682732 | 1.248847686 | 0.026159968 | 0.64842121 | ESI+ |
| 80 | Dimethyl sulfone | 226.98523 | 1.244080274 | 0.019160401 | 0.763937282 | ESI+ |
| 81 | Dihydroxyfumarate | 149.078854 | 1.170022703 | 0.012095579 | 0.488802041 | ESI+ |
| 82 | d-Erythro-sphingosine-1-phosphate | 380.2532053 | 1.737724467 | 0.000544924 | 0.596305495 | ESI+ |
| 83 | Deoxyinosine | 253.091054 | 1.613104511 | 0.005920452 | 0.545997619 | ESI+ |
| 84 | Decanoyl-l-carnitine | 393.1663611 | 1.425308463 | 0.016586775 | 0.510589347 | ESI+ |
| 85 | Daidzin | 417.1143251 | 1.175574074 | 0.034375716 | 0.502007087 | ESI+ |
| 86 | Cytosine | 112.0486335 | 1.420559475 | 0.007593508 | 0.670244653 | ESI+ |
| 87 | Cytidine | 509.1566932 | 1.052530835 | 0.028853049 | 0.601165436 | ESI+ |
| 88 | Creatinine | 114.0642295 | 1.388999521 | 0.003376307 | 0.699636566 | ESI+ |
| 89 | Coumarin | 147.0413483 | 1.430714045 | 0.003108357 | 0.742905612 | ESI+ |
| 90 | Choline | 104.1050474 | 1.177318332 | 0.043537875 | 0.757184059 | ESI+ |
| 91 | Chenodeoxycholate | 375.2864869 | 1.342769461 | 0.019219803 | 0.50993605 | ESI+ |
| 92 | Carbamazepine | 201.0842756 | 1.452874391 | 0.016478139 | 0.702864245 | ESI+ |
| 93 | Caproic acid | 158.1151287 | 1.145702051 | 0.042053088 | 0.694832662 | ESI+ |
| 94 | Biopterin | 238.0903409 | 1.384772691 | 0.00596766 | 0.712372868 | ESI+ |
| 95 | Behenic acid | 358.3653515 | 1.29565391 | 0.036821959 | 0.36862125 | ESI+ |
| 96 | Arg-Cys | 319.1472496 | 1.080342543 | 0.028692342 | 0.480205495 | ESI+ |
| 97 | Ala-Lys | 218.1553264 | 1.675919183 | 0.008837507 | 0.463916122 | ESI+ |
| 98 | 5-Methylcytidine | 318.1271949 | 1.126791265 | 0.029136132 | 0.709406549 | ESI+ |
| 99 | 5-Methyl-5,6-dihydrouracil | 279.10756 | 1.610141786 | 0.004509134 | 0.667621148 | ESI+ |
| 100 | 5-Methyl-2-thiouridine | 316.0979441 | 1.264486126 | 0.014961713 | 0.266099361 | ESI+ |
| 101 | 5-Hydroxyindoleacetate | 192.0662465 | 1.059345594 | 0.026283885 | 0.56081346 | ESI+ |
| 102 | 4-Acetamidobutanoate | 146.0787893 | 1.13653669 | 0.006568675 | 0.495555787 | ESI+ |
| 103 | 3'-O-Methylinosine | 305.0826964 | 1.30435505 | 0.009637954 | 0.682547669 | ESI+ |
| 104 | 3-Methylglutaric acid | 188.088954 | 1.485105926 | 0.009842965 | 0.389280251 | ESI+ |
| 105 | 3-Hydroxyphenylacetic acid | 153.0574779 | 1.58007533 | 0.000445244 | 0.420413666 | ESI+ |
| 106 | 3-Butynoic acid | 102.0527878 | 1.541924678 | 0.011313147 | 0.586920507 | ESI+ |
| 107 | 2'-O-methylcytidine | 299.1344078 | 1.467138394 | 0.00469822 | 0.630703057 | ESI+ |
| 108 | 2-Hydroxyadenine | 190.0099047 | 1.620432964 | 0.002870532 | 0.388255341 | ESI+ |
| 109 | 1-Stearoyl-2-hydroxy-sn-glycero-3-phosphocholine | 524.3667812 | 1.236039285 | 0.006997294 | 0.723468376 | ESI+ |
| 110 | 1-Palmitoyl-sn-glycero-3-phosphocholine | 496.3364584 | 1.312679903 | 0.008844814 | 0.683195757 | ESI+ |
| 111 | 1-Palmitoyllysophosphatidylcholine | 538.3833373 | 1.143184646 | 0.0144407 | 0.728003828 | ESI+ |
| 112 | 1-O-Octadecyl-sn-glyceryl-3-phosphorylcholine | 509.3768663 | 1.241799183 | 0.012566425 | 0.647749204 | ESI+ |
| 113 | 1-Oleoyl-sn-glycero-3-phosphocholine | 522.3507632 | 1.1978656 | 0.025090387 | 0.662150775 | ESI+ |
| 114 | 1-O-(cis-9-Octadecenyl)-2-O-acetyl-sn-glycero-3-phosphocholine | 550.3824178 | 1.342060588 | 0.011836055 | 0.642025574 | ESI+ |
| 115 | 1-Myristoyl-sn-glycero-3-phosphocholine | 468.3064395 | 1.358576117 | 0.01975004 | 0.587560686 | ESI+ |
| 116 | 1-Methylguanosine | 298.1112803 | 1.030886988 | 0.031510676 | 0.747568637 | ESI+ |
| 117 | 1-Aminocyclohexanecarboxylic acid | 144.0990458 | 1.220593326 | 0.032542441 | 0.639004346 | ESI+ |
| 118 | (S)-2-Hydroxyglutarate | 319.0617979 | 1.386089757 | 0.013324826 | 0.718352742 | ESI+ |
| 119 | (3-Carboxypropyl)trimethylammonium cation | 146.1155281 | 1.446942061 | 0.014707039 | 0.641245481 | ESI+ |
| 120 | (-)-Medicarpin | 270.0888945 | 1.203058055 | 0.016581814 | 0.465737845 | ESI+ |
| 121 | Vanillin | 173.0252444 | 1.363542696 | 0.035368961 | 0.694782222 | ESI− |
| 122 | Uric acid | 167.019733 | 1.92536798 | 0.001591207 | 0.359290578 | ESI− |
| 123 | Uracil | 111.020074 | 1.420172951 | 0.016153532 | 0.633118434 | ESI− |
| 124 | Tryptamine | 219.1127183 | 1.284826608 | 0.040679251 | 0.39129697 | ESI− |
| 125 | Tetracosanoic acid | 367.3547167 | 1.081796548 | 0.047411231 | 0.483273704 | ESI− |
| 126 | Suberylglycine | 290.1225713 | 1.252652972 | 0.017491287 | 0.374856891 | ESI− |
| 127 | Stavudine | 223.0716647 | 1.4889081 | 0.009050235 | 0.613851018 | ESI− |
| 128 | Sphingosine-1-phosphate | 378.2394404 | 1.638049055 | 0.002718586 | 0.619737134 | ESI− |
| 129 | Sarcosine | 88.03981469 | 1.551541097 | 0.011620552 | 0.646903454 | ESI− |
| 130 | Salidroside | 281.1056812 | 1.073901898 | 0.018778697 | 0.40522896 | ESI− |
| 131 | Ribulose 5-phosphate | 289.0372061 | 1.783397494 | 0.002898077 | 0.552258752 | ESI− |
| 132 | Ribothymidine | 279.054457 | 1.600838378 | 0.013582127 | 0.628447063 | ESI− |
| 133 | Primidone | 234.1236793 | 1.796265036 | 0.006889804 | 0.346056324 | ESI− |
| 134 | p-Hydroxycinnamaldehyde | 185.0011304 | 1.922669684 | 0.001624933 | 0.350537556 | ESI− |
| 135 | Phosphatidylinositol | 865.5794594 | 1.290049585 | 0.009809233 | 0.733895683 | ESI− |
| 136 | Phenyllactic acid | 165.0548945 | 1.691483934 | 0.01532848 | 0.517972434 | ESI− |
| 137 | Phenethyl Caffeiate | 321.0479463 | 1.250965689 | 0.015393657 | 0.439522151 | ESI− |
| 138 | Perseitol | 193.070691 | 1.821766989 | 0.009080271 | 0.538453929 | ESI− |
| 139 | Pentobarbital | 225.1235408 | 1.504447344 | 0.043583282 | 0.657993809 | ESI− |
| 140 | Pectin (galacturonic acid) | 193.0341871 | 1.193771726 | 0.044851158 | 0.787078208 | ESI− |
| 141 | Pantothenate | 218.1026502 | 1.562562874 | 0.010070368 | 0.587744773 | ESI− |
| 142 | Oxypurinol | 133.0145843 | 1.963472783 | 0.000318001 | 0.324896866 | ESI− |
| 143 | *O*-Phospho-l-threonine | 199.0213922 | 1.301404372 | 0.045814054 | 0.510264303 | ESI− |
| 144 | Norethindrone acetate | 339.1977445 | 1.271639853 | 0.020686515 | 0.683496831 | ESI− |
| 145 | Nicotinamide ribotide | 333.0571167 | 1.149136627 | 0.027362389 | 0.548548029 | ESI− |
| 146 | *N*-Formylmethionine | 176.0373757 | 1.835429574 | 0.013913348 | 0.403626091 | ESI− |
| 147 | *N*-Acetylneuraminic acid | 309.1002023 | 1.301130885 | 0.040240786 | 0.710393224 | ESI− |
| 148 | *N*-Acetyl-l-phenylalanine | 206.0813136 | 1.42856151 | 0.00710991 | 0.558850242 | ESI− |
| 149 | *N*-Acetyl-l-aspartic acid | 174.0398781 | 1.268777715 | 0.010690718 | 0.555172933 | ESI− |
| 150 | *N*-Acetyl-d-lactosamine | 399.1667469 | 1.382441 | 0.029308216 | 0.424521747 | ESI− |
| 151 | *N*-Acetyl-d-glucosamine 6-phosphate | 301.0558673 | 1.551934718 | 0.023815613 | 0.710833248 | ESI− |
| 152 | *N*2-Acetyl-l-ornithine | 173.0920188 | 1.664701011 | 0.002378318 | 0.678898128 | ESI− |
| 153 | Myristoleic acid | 225.1852283 | 1.257775683 | 0.025759169 | 0.513506216 | ESI− |
| 154 | Monomethyl glutaric acid | 162.0760105 | 1.605152798 | 0.006376857 | 0.662341424 | ESI− |
| 155 | Mesaconic acid | 129.0183686 | 1.483859702 | 0.010913936 | 0.58330637 | ESI− |
| 156 | m-Chlorohippuric acid | 212.0110696 | 1.373525973 | 0.043806319 | 0.58149853 | ESI− |
| 157 | Malonic acid | 103.0028384 | 1.516423474 | 0.006691611 | 0.600447545 | ESI− |
| 158 | Maleamic acid | 174.0401038 | 1.269554236 | 0.024679581 | 0.582342963 | ESI− |
| 159 | l-Valine | 116.0712548 | 1.454218176 | 0.019828333 | 0.702431317 | ESI− |
| 160 | l-Threonate | 135.029438 | 1.184224693 | 0.024731642 | 0.647540584 | ESI− |
| 161 | l-Pyroglutamic acid | 257.0763492 | 1.094190693 | 0.032209614 | 0.507904337 | ESI− |
| 162 | l-Homocysteic acid | 199.0366447 | 1.50051889 | 0.011618307 | 0.607132758 | ESI− |
| 163 | Ketoisocaproic acid | 129.0552399 | 1.816503286 | 0.014928085 | 0.113265456 | ESI− |
| 164 | Indoxyl sulfate | 212.0010915 | 1.26580141 | 0.038773415 | 0.61523562 | ESI− |
| 165 | Indolelactic acid | 204.0655531 | 1.506952659 | 0.015307451 | 0.637725828 | ESI− |
| 166 | Indoleacetic acid | 174.058934 | 1.684675908 | 0.009935795 | 0.587816871 | ESI− |
| 167 | Indole | 176.0708415 | 1.623950126 | 0.040322984 | 0.42707711 | ESI− |
| 168 | Hydroxyisocaproic acid | 131.0705556 | 1.470632458 | 0.008212656 | 0.553066535 | ESI− |
| 169 | Hydrocortisone 21-acetate | 385.2105293 | 1.434622007 | 0.023576691 | 0.484316327 | ESI− |
| 170 | Glycine | 74.02469357 | 1.445747809 | 0.006827488 | 0.758209366 | ESI− |
| 171 | Glycerol 3-phosphate | 152.995061 | 1.172204933 | 0.046443072 | 0.678551324 | ESI− |
| 172 | Glyceric acid | 165.039304 | 1.06035919 | 0.03110135 | 0.627314102 | ESI− |
| 173 | Glafenine | 431.1094498 | 1.750721989 | 0.001219612 | 0.664235749 | ESI− |
| 174 | γ-l-Glutamyl-l-phenylalanine | 353.1428505 | 1.314785558 | 0.041517888 | 0.696152496 | ESI− |
| 175 | γ-l-Glutamyl-l-glutamic acid | 335.1079344 | 1.211319065 | 0.017180908 | 0.504329115 | ESI− |
| 176 | Galangin | 269.0517146 | 1.031784543 | 0.046385512 | 0.686684412 | ESI− |
| 177 | d-Sorbitol | 203.0549992 | 1.248241081 | 0.020544255 | 0.216617183 | ESI− |
| 178 | dl-Methionine sulfoxide | 164.0375235 | 1.93618885 | 0.002276798 | 0.517666514 | ESI− |
| 179 | dl-Lactate | 89.02452651 | 1.383175486 | 0.038390687 | 0.648914585 | ESI− |
| 180 | Dioxybenzone | 225.0502924 | 1.344073276 | 0.035368793 | 0.55805431 | ESI− |
| 181 | Dihydroxyacetone phosphate | 229.0102608 | 1.064429442 | 0.039886334 | 0.682732234 | ESI− |
| 182 | Dihydrouracil | 173.0559334 | 1.7470294 | 0.005546336 | 0.548132594 | ESI− |
| 183 | Dicumarol | 352.0854024 | 1.54332714 | 0.034508597 | 0.664643964 | ESI− |
| 184 | d-Glucuronate | 387.0698882 | 1.080798385 | 0.003598399 | 0.350176828 | ESI− |
| 185 | d-Fructose | 179.055091 | 1.437899019 | 0.030911255 | 0.57941308 | ESI− |
| 186 | d-Biotin | 243.0800983 | 1.686880805 | 0.00158561 | 0.648344055 | ESI− |
| 187 | d(-)-beta-hydroxy butyric acid | 163.0602347 | 1.385051726 | 0.019780043 | 0.621330259 | ESI− |
| 188 | Cortisone acetate | 401.198305 | 1.607633104 | 0.00695664 | 0.560009003 | ESI− |
| 189 | Buprenorphine | 466.2922691 | 1.33848972 | 0.019927867 | 0.723799882 | ESI− |
| 190 | Bufexamac | 244.0919363 | 1.163616644 | 0.029447088 | 0.740658748 | ESI− |
| 191 | Benzoic acid | 121.0289104 | 1.296799898 | 0.028306136 | 0.621783441 | ESI− |
| 192 | Ammelide | 128.0346964 | 1.395593347 | 0.043706909 | 0.858341332 | ESI− |
| 193 | Alpha-ketoisovaleric acid | 115.0396748 | 1.839273005 | 0.011162906 | 0.125668676 | ESI− |
| 194 | Alpha-ketoglutarate | 145.0135977 | 1.929028411 | 0.003289809 | 0.193417578 | ESI− |
| 195 | Allantoate/allantoic acid | 175.0429933 | 1.248587211 | 0.022552679 | 0.624292949 | ESI− |
| 196 | Adenosine | 248.0790291 | 1.396933619 | 0.022082034 | 0.76604673 | ESI− |
| 197 | Aconitic acid | 173.0078074 | 1.384967109 | 0.017262454 | 0.586440041 | ESI− |
| 198 | Acetylglycine | 116.0346083 | 1.544467085 | 0.009688018 | 0.668883969 | ESI− |
| 199 | 3R-hydroxy-butanoic acid | 125.0235979 | 1.146888045 | 0.027597806 | 0.703322177 | ESI− |
| 200 | 3-Methylhistidine | 168.0770513 | 1.225992943 | 0.028864254 | 0.798386282 | ESI− |
| 201 | 3-Methylhistamine | 146.0715347 | 1.679955799 | 0.020782572 | 0.306451871 | ESI− |
| 202 | 3-Hydroxyisovaleric acid | 99.04415609 | 1.265339362 | 0.038294395 | 0.763540618 | ESI− |
| 203 | 2-Oxoadipic acid | 141.0166001 | 1.44476156 | 0.015589075 | 0.752779444 | ESI− |
| 204 | 2'-O-Methylinosine | 281.0876016 | 1.386357811 | 0.024093683 | 0.694364224 | ESI− |
| 205 | 2-Methylbenzoic acid | 135.0444613 | 1.309123042 | 0.007530434 | 0.41744307 | ESI− |
| 206 | 2-Hydroxy-butanoic acid | 103.0396367 | 1.586283379 | 0.003506274 | 0.612543931 | ESI− |
| 207 | 2'-Deoxyuridine | 227.0667242 | 1.656340531 | 0.003403923 | 0.604605058 | ESI− |
| 208 | 2-Deoxyribose 5-phosphate | 273.0421359 | 1.547033474 | 0.021645587 | 0.247759082 | ESI− |
| 209 | 2-Dehydro-3-deoxy-d-gluconate | 177.039347 | 1.240662929 | 0.019474021 | 0.646093263 | ESI− |
| 210 | 1-Naphthol | 143.0495746 | 1.062565335 | 0.022752997 | 0.566034717 | ESI− |
| 211 | 1-Methyladenosine | 302.0824436 | 1.000244154 | 0.016876852 | 0.469060923 | ESI− |
| 212 | 1,2-Benzenedicarboxylic acid | 165.0198166 | 1.716300761 | 0.009399358 | 0.610833214 | ESI− |

MS2, secondary mass spectrometry; m/z, mass-to-charge ratio; VIP, variable importance in projection; ESI, electrospray ionization.

**Table S4.** Differential metabolites in *Clonorchis sinensis*-infected rabbit at 28 days post-infection.

| ID | MS2 name | m/z | VIP | *P*-Value | Fold change | ESI |
| --- | --- | --- | --- | --- | --- | --- |
| 1 | Xanthine | 153.0380672 | 1.363778051 | 0.0454508 | 0.625328849 | ESI+ |
| 2 | Val-Arg | 274.1924919 | 2.461059403 | 0.010456727 | 0.607238791 | ESI+ |
| 3 | Uridine | 245.0740259 | 2.179058836 | 0.018265986 | 1.611419673 | ESI+ |
| 4 | Uracil | 113.0324411 | 2.160989277 | 0.025010449 | 1.730568176 | ESI+ |
| 5 | Phytosphingosine | 282.2762903 | 1.98163107 | 0.016758417 | 1.430557879 | ESI+ |
| 6 | *N*-Acetyl-l-glutamic acid | 231.0950253 | 1.889959411 | 0.032220773 | 1.317110612 | ESI+ |
| 7 | Glycodeoxycholic acid | 450.3182546 | 2.593573916 | 0.034041819 | 2.930717107 | ESI+ |
| 8 | Cyclohexylamine | 141.136023 | 1.753315763 | 0.047824279 | 0.816143344 | ESI+ |
| 9 | Acetyl tyrosine ethyl ester | 315.1315498 | 2.126917673 | 0.02218718 | 2.303487901 | ESI+ |
| 10 | 3-Hydroxyphenylacetic acid | 153.0574779 | 2.301916308 | 0.014581794 | 0.536372908 | ESI+ |
| 11 | 2-Hydroxyadenine | 190.0099047 | 2.365079715 | 0.007936865 | 0.40642964 | ESI+ |
| 12 | 1-Oleoyl-sn-glycerol 3-phosphate | 437.2618854 | 2.384384656 | 0.026934997 | 2.062900667 | ESI+ |
| 13 | (Z)-6-Octadecenoic acid | 300.2867683 | 2.395538277 | 0.005298669 | 1.558750811 | ESI+ |
| 14 | 1-Oleoyl-l-α-lysophosphatidic acid | 435.2487764 | 1.969841306 | 0.030798854 | 2.043540212 | ESI− |
| 15 | 1-Palmitoyl lysophosphatidic acid | 409.2334892 | 2.000037516 | 0.028385312 | 2.203203422 | ESI− |
| 16 | 4-Hydroxycinnamic acid | 223.0652764 | 2.696193669 | 0.005926156 | 0.387979136 | ESI− |
| 17 | Adynerin | 515.3034999 | 1.182347759 | 0.019574466 | 1.584484454 | ESI− |
| 18 | d-Biotin | 243.0800983 | 1.807227255 | 0.042143822 | 0.752896193 | ESI− |
| 19 | Glycolithocholic acid | 432.3095992 | 3.066501804 | 0.018909706 | 2.663748922 | ESI− |
| 20 | l-Pipecolic acid | 128.0709112 | 2.133070519 | 0.010386417 | 1.935095409 | ESI− |
| 21 | l-Tryptophan | 203.082121 | 1.166516638 | 0.034588025 | 0.781020732 | ESI− |
| 22 | Oxypurinol | 133.0145843 | 2.296319354 | 0.035030382 | 0.606027425 | ESI− |
| 23 | Taurochenodeoxycholate | 498.2862366 | 2.522681731 | 0.042669928 | 2.502713269 | ESI− |

MS2, secondary mass spectrometry; m/z, mass-to-charge ratio; VIP, variable importance in projection; ESI, electrospray ionization.

**Table S5.** Differential metabolites in *Clonorchis sinensis*-infected rabbit at 63 days post-infection.

| ID | MS2 name | m/z | VIP | *P*-Value | Fold change | ESI |
| --- | --- | --- | --- | --- | --- | --- |
| 1 | Xanthine | 153.0380672 | 2.471899937 | 0.008295579 | 0.532670697 | ESI+ |
| 2 | Val-Arg | 274.1924919 | 2.116081984 | 0.025598554 | 0.642062545 | ESI+ |
| 3 | Stearidonic Acid | 277.2136447 | 2.422853914 | 0.00844462 | 0.71835032 | ESI+ |
| 4 | Nicotinamide N-oxide | 139.0480076 | 1.847076064 | 0.039024334 | 0.774559992 | ESI+ |
| 5 | N2-Acetyl-L-ornithine | 175.1051007 | 2.03625691 | 0.047114103 | 0.766020833 | ESI+ |
| 6 | Methylmalonic acid | 119.0327104 | 1.833519846 | 0.036503426 | 0.605777732 | ESI+ |
| 7 | L-Tryptophan | 205.0942531 | 1.8111358 | 0.038653535 | 0.754244446 | ESI+ |
| 8 | L-Isoleucine | 173.1255799 | 2.37408493 | 0.021891049 | 1.448069436 | ESI+ |
| 9 | Ile-Phe | 557.3217124 | 2.485778079 | 0.048929264 | 2.962951099 | ESI+ |
| 10 | Gly-Glu | 246.1058104 | 2.318544398 | 0.045144098 | 1.773013422 | ESI+ |
| 11 | dl-O-tyrosine | 146.0573407 | 1.988752854 | 0.036282283 | 0.811878073 | ESI+ |
| 12 | dl-Indole-3-lactic acid | 188.0682089 | 1.981958 | 0.024095419 | 0.748871482 | ESI+ |
| 13 | d-erythro-Sphingosine-1-phosphate | 380.2532053 | 2.1435594 | 0.027440791 | 0.785872782 | ESI+ |
| 14 | l-Pipecolic acid | 128.0709112 | 1.666833038 | 0.027360604 | 1.739703586 | ESI− |
| 15 | Hypoxanthine | 135.0310739 | 2.236919137 | 0.041839426 | 0.628045999 | ESI− |
| 16 | Hexadecanedioic acid | 285.2054278 | 2.787777022 | 0.009550883 | 0.678190986 | ESI− |
| 17 | Glycolithocholic acid | 432.3095992 | 3.096015842 | 0.039235515 | 3.057258165 | ESI− |
| 18 | Glycodeoxycholic acid | 448.3059647 | 2.535900887 | 0.024834519 | 3.358508581 | ESI− |
| 19 | dl-Mandelic acid | 151.0392365 | 2.780546961 | 0.015255142 | 0.594476174 | ESI− |
| 20 | 3-Deoxy-2-keto-6-phosphogluconic acid | 317.1268206 | 1.819763668 | 0.047055455 | 0.673601792 | ESI− |
| 21 | (R)-Mevalonic acid 5-phosphate | 209.1171275 | 2.083606558 | 0.022945007 | 0.639804223 | ESI− |

MS2, secondary mass spectrometry; m/z, mass-to-charge ratio; VIP, variable importance in projection; ESI, electrospray ionization.

**Tables S6.** Metabolic pathways in different *Clonorchis sinensis* infection phases.

| Pathways | Total metabolites | Hit metabolites | Raw p | -ln(p) | FDR | Impact |
| --- | --- | --- | --- | --- | --- | --- |
| 7 dpi |  |  |  |  |  |  |
| Pyrimidine metabolism | 60 | 8 | 2.0324E-06 | 13.106 | 0.00016259 | 0.19879 |
| Nitrogen metabolism | 39 | 4 | 0.0026475 | 5.9341 | 0.1059 | 0.00763 |
| Aminoacyl-tRNA biosynthesis | 75 | 5 | 0.0051347 | 5.2717 | 0.11496 | 0 |
| Arginine and proline metabolism | 77 | 5 | 0.0057482 | 5.1589 | 0.11496 | 0.11972 |
| Cyanoamino acid metabolism | 16 | 2 | 0.024099 | 3.7256 | 0.38558 | 0 |
| Phenylalanine metabolism | 45 | 3 | 0.030467 | 3.4911 | 0.40623 | 0.1606 |
| Glycine, serine and threonine metabolism | 48 | 3 | 0.035989 | 3.3245 | 0.4113 | 0.23804 |
| Alanine, aspartate and glutamate metabolism | 24 | 2 | 0.051335 | 2.9694 | 0.51335 | 0.25261 |
| Pantothenate and CoA biosynthesis | 27 | 2 | 0.063444 | 2.7576 | 0.54142 | 0.07366 |
| beta-Alanine metabolism | 28 | 2 | 0.067678 | 2.693 | 0.54142 | 0 |
| d-Arginine and d-ornithine metabolism | 8 | 1 | 0.11672 | 2.148 | 0.83122 | 0.5 |
| Valine, leucine and isoleucine degradation | 40 | 2 | 0.12468 | 2.082 | 0.83122 | 0.01657 |
| Nicotinate and nicotinamide metabolism | 44 | 2 | 0.14566 | 1.9265 | 0.8632 | 0.08613 |
| d-Glutamine and d-Glutamate metabolism | 11 | 1 | 0.15698 | 1.8517 | 0.8632 | 0.02674 |
| Lysine degradation | 47 | 2 | 0.16185 | 1.8211 | 0.8632 | 0.0121 |
| Thiamine metabolism | 24 | 1 | 0.31174 | 1.1656 | 1 | 0 |
| Valine, leucine and isoleucine biosynthesis | 27 | 1 | 0.34332 | 1.0691 | 1 | 0.0885 |
| Pentose phosphate pathway | 32 | 1 | 0.39284 | 0.93435 | 1 | 0 |
| Pyruvate metabolism | 32 | 1 | 0.39284 | 0.93435 | 1 | 0.05702 |
| Lysine biosynthesis | 32 | 1 | 0.39284 | 0.93435 | 1 | 0.05875 |
| Methane metabolism | 34 | 1 | 0.41162 | 0.88767 | 1 | 0 |
| Purine metabolism | 92 | 2 | 0.41685 | 0.87503 | 1 | 0 |
| Propanoate metabolism | 35 | 1 | 0.42079 | 0.86562 | 1 | 0.0003 |
| Glutathione metabolism | 38 | 1 | 0.44748 | 0.80411 | 1 | 0 |
| Histidine metabolism | 44 | 1 | 0.49733 | 0.6985 | 1 | 0.13988 |
| Primary bile acid biosynthesis | 47 | 1 | 0.52059 | 0.6528 | 1 | 0.00822 |
| Glyoxylate and dicarboxylate metabolism | 50 | 1 | 0.54279 | 0.61103 | 1 | 0.02569 |
| Cysteine and methionine metabolism | 56 | 1 | 0.58424 | 0.53744 | 1 | 0.0478 |
| Tyrosine metabolism | 76 | 1 | 0.69766 | 0.36002 | 1 | 0.07227 |
| Tryptophan metabolism | 79 | 1 | 0.71184 | 0.3399 | 1 | 0.027 |
| Amino sugar and nucleotide sugar metabolism | 88 | 1 | 0.7506 | 0.28689 | 1 | 0.02488 |
| Porphyrin and chlorophyll metabolism | 104 | 1 | 0.80735 | 0.214 | 1 | 0 |
| 14 dpi |  |  |  |  |  |  |
| Glycine, serine and threonine metabolism | 48 | 11 | 0.000017882 | 10.932 | 0.0014306 | 0.51063 |
| Aminoacyl-tRNA biosynthesis | 75 | 13 | 0.000072797 | 9.5278 | 0.0029119 | 0.16902 |
| Arginine and proline metabolism | 77 | 11 | 0.0014847 | 6.5126 | 0.038471 | 0.21824 |
| Pantothenate and CoA biosynthesis | 27 | 6 | 0.0019235 | 6.2536 | 0.038471 | 0.27382 |
| Pyrimidine metabolism | 60 | 9 | 0.0028639 | 5.8556 | 0.045822 | 0.17441 |
| Phenylalanine metabolism | 45 | 7 | 0.0068651 | 4.9813 | 0.091535 | 0.14335 |
| Valine, leucine and isoleucine biosynthesis | 27 | 5 | 0.01047 | 4.5593 | 0.11965 | 0.14044 |
| beta-Alanine metabolism | 28 | 5 | 0.012236 | 4.4034 | 0.12236 | 0.03447 |
| d-Glutamine and d-Glutamate metabolism | 11 | 3 | 0.015892 | 4.1419 | 0.13674 | 0.13904 |
| Purine metabolism | 92 | 10 | 0.017093 | 4.0691 | 0.13674 | 0.07667 |
| Alanine, aspartate and glutamate metabolism | 24 | 4 | 0.031169 | 3.4683 | 0.22668 | 0.38367 |
| Nitrogen metabolism | 39 | 5 | 0.045887 | 3.0816 | 0.28238 | 0 |
| Glycerophospholipid metabolism | 39 | 5 | 0.045887 | 3.0816 | 0.28238 | 0.0636 |
| Valine, leucine and isoleucine degradation | 40 | 5 | 0.050375 | 2.9883 | 0.28786 | 0.08099 |
| Sulfur metabolism | 18 | 3 | 0.060604 | 2.8034 | 0.32322 | 0.07205 |
| Biotin metabolism | 11 | 2 | 0.10537 | 2.2502 | 0.52687 | 0.20325 |
| Thiamine metabolism | 24 | 3 | 0.12078 | 2.1138 | 0.56014 | 0 |
| Glutathione metabolism | 38 | 4 | 0.12603 | 2.0712 | 0.56014 | 0.01285 |
| Cyanoamino acid metabolism | 16 | 2 | 0.19541 | 1.6326 | 0.77371 | 0 |
| Tryptophan metabolism | 79 | 6 | 0.21386 | 1.5424 | 0.77371 | 0.11598 |
| Glycerolipid metabolism | 32 | 3 | 0.22244 | 1.5031 | 0.77371 | 0.02774 |
| Lysine biosynthesis | 32 | 3 | 0.22244 | 1.5031 | 0.77371 | 0.15868 |
| Pentose phosphate pathway | 32 | 3 | 0.22244 | 1.5031 | 0.77371 | 0.16269 |
| Glyoxylate and dicarboxylate metabolism | 50 | 4 | 0.25048 | 1.3844 | 0.83494 | 0.06177 |
| Propanoate metabolism | 35 | 3 | 0.26421 | 1.331 | 0.83823 | 0.0003 |
| Citrate cycle (TCA cycle) | 20 | 2 | 0.27243 | 1.3004 | 0.83823 | 0.14088 |
| Caffeine metabolism | 21 | 2 | 0.29179 | 1.2317 | 0.86455 | 0.0305 |
| Cysteine and methionine metabolism | 56 | 4 | 0.32041 | 1.1382 | 0.88596 | 0.17877 |
| Inositol phosphate metabolism | 39 | 3 | 0.32116 | 1.1358 | 0.88596 | 0.05887 |
| D-Arginine and D-ornithine metabolism | 8 | 1 | 0.34312 | 1.0697 | 0.91498 | 0.5 |
| Sphingolipid metabolism | 25 | 2 | 0.36818 | 0.99917 | 0.95015 | 0.02575 |
| Nicotinate and nicotinamide metabolism | 44 | 3 | 0.39245 | 0.93535 | 0.95139 | 0.08613 |
| Histidine metabolism | 44 | 3 | 0.39245 | 0.93535 | 0.95139 | 0.14039 |
| Phenylalanine, tyrosine and tryptophan biosynthesis | 27 | 2 | 0.40525 | 0.90326 | 0.95352 | 0.00738 |
| Lysine degradation | 47 | 3 | 0.43441 | 0.83377 | 0.99293 | 0.15885 |
| Fatty acid biosynthesis | 49 | 3 | 0.4618 | 0.77261 | 1 | 0 |
| Pyruvate metabolism | 32 | 2 | 0.49297 | 0.70731 | 1 | 0.05702 |
| Pentose and glucuronate interconversions | 53 | 3 | 0.5148 | 0.66397 | 1 | 0.14645 |
| Glycosylphosphatidylinositol(GPI)-anchor biosynthesis | 14 | 1 | 0.52116 | 0.6517 | 1 | 0 |
| Methane metabolism | 34 | 2 | 0.52574 | 0.64295 | 1 | 0.01751 |
| Linoleic acid metabolism | 15 | 1 | 0.54577 | 0.60556 | 1 | 0.65625 |
| Tyrosine metabolism | 76 | 4 | 0.5509 | 0.5962 | 1 | 0.04743 |
| Butanoate metabolism | 40 | 2 | 0.61529 | 0.48566 | 1 | 0 |
| Galactose metabolism | 41 | 2 | 0.6289 | 0.46378 | 1 | 0 |
| Taurine and hypotaurine metabolism | 20 | 1 | 0.65123 | 0.4289 | 1 | 0 |
| Ascorbate and aldarate metabolism | 45 | 2 | 0.67966 | 0.38616 | 1 | 0.03322 |
| Selenoamino acid metabolism | 22 | 1 | 0.68625 | 0.37651 | 1 | 0.00482 |
| Ether lipid metabolism | 23 | 1 | 0.70243 | 0.35321 | 1 | 0 |
| Primary bile acid biosynthesis | 47 | 2 | 0.70286 | 0.35259 | 1 | 0.00849 |
| Fructose and mannose metabolism | 48 | 2 | 0.71394 | 0.33696 | 1 | 0.05002 |
| Starch and sucrose metabolism | 50 | 2 | 0.73506 | 0.30781 | 1 | 0 |
| Fatty acid elongation in mitochondria | 27 | 1 | 0.75928 | 0.27538 | 1 | 0 |
| Glycolysis or Gluconeogenesis | 31 | 1 | 0.80535 | 0.21648 | 1 | 0 |
| Vitamin B6 metabolism | 32 | 1 | 0.81542 | 0.20405 | 1 | 0.01914 |
| Amino sugar and nucleotide sugar metabolism | 88 | 3 | 0.8385 | 0.17614 | 1 | 0.01426 |
| Ubiquinone and other terpenoid-quinone biosynthesis | 36 | 1 | 0.85081 | 0.16157 | 1 | 0 |
| Porphyrin and chlorophyll metabolism | 104 | 3 | 0.91026 | 0.094025 | 1 | 0 |
| Fatty acid metabolism | 50 | 1 | 0.92938 | 0.073238 | 1 | 0.02959 |
| Drug metabolism - cytochrome P450 | 86 | 1 | 0.9899 | 0.010156 | 1 | 0.03519 |
| 28 dpi |  |  |  |  |  |  |
| Phenylalanine metabolism | 45 | 2 | 0.026969 | 3.6131 | 1 | 0.01193 |
| Pyrimidine metabolism | 60 | 2 | 0.045881 | 3.0817 | 1 | 0.09193 |
| Biotin metabolism | 11 | 1 | 0.062277 | 2.7762 | 1 | 0.20325 |
| Caffeine metabolism | 21 | 1 | 0.11575 | 2.1563 | 1 | 0.0305 |
| Sphingolipid metabolism | 25 | 1 | 0.13633 | 1.9927 | 1 | 0 |
| Phenylalanine, tyrosine and tryptophan biosynthesis | 27 | 1 | 0.14646 | 1.921 | 1 | 0 |
| Pantothenate and CoA biosynthesis | 27 | 1 | 0.14646 | 1.921 | 1 | 0 |
| beta-Alanine metabolism | 28 | 1 | 0.15148 | 1.8873 | 1 | 0 |
| Ubiquinone and other terpenoid-quinone biosynthesis | 36 | 1 | 0.19067 | 1.6572 | 1 | 0.0337 |
| Nitrogen metabolism | 39 | 1 | 0.20493 | 1.5851 | 1 | 0 |
| Primary bile acid biosynthesis | 47 | 1 | 0.24182 | 1.4196 | 1 | 0.00992 |
| Glycine, serine and threonine metabolism | 48 | 1 | 0.24631 | 1.4011 | 1 | 0 |
| Aminoacyl-tRNA biosynthesis | 75 | 1 | 0.35878 | 1.025 | 1 | 0 |
| Tyrosine metabolism | 76 | 1 | 0.36263 | 1.0144 | 1 | 0 |
| Arginine and proline metabolism | 77 | 1 | 0.36646 | 1.0039 | 1 | 0.00923 |
| Tryptophan metabolism | 79 | 1 | 0.37405 | 0.98336 | 1 | 0.10853 |
| Purine metabolism | 92 | 1 | 0.42138 | 0.86422 | 1 | 0.03617 |
| 63 dpi |  |  |  |  |  |  |
| Valine, leucine and isoleucine degradation | 40 | 2 | 0.021603 | 3.8349 | 1 | 0 |
| Glycine, serine and threonine metabolism | 48 | 2 | 0.030424 | 3.4925 | 1 | 0.02337 |
| Aminoacyl-tRNA biosynthesis | 75 | 2 | 0.068453 | 2.6816 | 1 | 0 |
| Purine metabolism | 92 | 2 | 0.097634 | 2.3265 | 1 | 0.04408 |
| Caffeine metabolism | 21 | 1 | 0.11575 | 2.1563 | 1 | 0.0305 |
| Sphingolipid metabolism | 25 | 1 | 0.13633 | 1.9927 | 1 | 0.02575 |
| Phenylalanine, tyrosine and tryptophan biosynthesis | 27 | 1 | 0.14646 | 1.921 | 1 | 0 |
| Valine, leucine and isoleucine biosynthesis | 27 | 1 | 0.14646 | 1.921 | 1 | 0.01325 |
| alpha-Linolenic acid metabolism | 29 | 1 | 0.15647 | 1.8549 | 1 | 0 |
| Terpenoid backbone biosynthesis | 33 | 1 | 0.17617 | 1.7363 | 1 | 0.04799 |
| Propanoate metabolism | 35 | 1 | 0.18586 | 1.6827 | 1 | 0.0003 |
| Nitrogen metabolism | 39 | 1 | 0.20493 | 1.5851 | 1 | 0 |
| Pyrimidine metabolism | 60 | 1 | 0.29839 | 1.2094 | 1 | 0 |
| Arginine and proline metabolism | 77 | 1 | 0.36646 | 1.0039 | 1 | 0.00447 |
| Tryptophan metabolism | 79 | 1 | 0.37405 | 0.98336 | 1 | 0.10853 |

dpi, days post-infection; FDR, false discover rate.
